# Supplementary material for: Transcriptomic Evidence Reveals the Dysfunctional Mechanism of Synaptic Plasticity Control in ASD
Source: Genes (Basel). 2024 Dec 25;16(1):11. doi: 10.3390/genes16010011 (PMC11764921; doi:10.3390/genes16010011)
Supplement: Supplementary file 1 [file genes-16-00011-s001.zip › Supplementary Figures and Tables.pdf]

Supplementary Information for

Convergent Transcriptomic Evidence Reveals the Dysfunctional Quantitative Mechanism of Synaptic Plasticity Control in ASD

Chao Kong, Zhi-Tong Bing, Lei Yang, Zi-Gang Huang, Wen-Xu Wang, and Celso Grebogi

Corresponding author: Zi-Gang Huang and Wen-Xu Wang

Contents

Supplementary Note 1 The Evidence for the Endophenotype of Synaptic Plasticity 1
- Synapse-related dysfunction feature by Gene enrichment analysis in ASD 1
  - Gene enrichment analysis 1
  - Progressive enrichment analysis in Reactome 1
  - Enrichment analysis in KEGG 1
- The relationship between cerebral cortex layers and glutamate neurons 1
- The relationship between ASD and intermediate neurons 2
- INs with LTP or LTD 2

Supplementary Note 2 The Pipeline of Constructing the mSiReN 2
- Initial nodes and final nodes in mSiReN 2
- Match with annotation database 3
- Match with interaction database 4

Supplementary Note 3 The NIVaCaR Algorithm 4
- Using FC value of DEGs to discover the common core module by NIVaCaR 6

Supplementary Note 4 The ProComReN Algorithm 6
- The mathematical representation of the ProComReN algorithm 7
- The relationship between PBN and DBN 8
- Assignment of additive effects to logic gates 9

Supplementary Note 5 The Pipeline of mSiReN Reduction for ProComReN Algorithm 11

Supplementary Note 6 Contextualizing mSiReN with ProComReN 12
- Establishing the experimental data-based quantitative model 12
  - Experiment-liked data to train the model 12
  - The preprocessing of data for the ProComReN model 12
  - Use the scRNA-seq expression data of CTL and ASD as the input of modeling 13
- Assessing discrepancies between two main cell types 13

Supplementary Note 7 Relationship between NIVaCaR and ProComReN 13
- General network structure and specific modeling 13
- The combinations of NIVaCaR and ProComReN 13
- The problem for core module-based inverse trace 14
- The limit of union network to train the model 14

Supplementary Note 8 The Significance of mSiReN in Investigating Dysfunctions in Signal Transduction 14
- Gene mutation, RNA, protein, and phenotypic effects 14
- The necessity of training quantitative model 15
- The advantages of mSiReN in investigating dysfunction mechanisms 15

Supplementary Figure 17

Supplementary Table 29

Supplementary Note S1 The Evidence for the Endophenotype of Synaptic Plasticity

Synapse-related dysfunction feature by Gene enrichment analysis in ASD

Gene enrichment analysis

Gene Enrichment Analysis (GEA) is a widely used approach to identifying biological connections. We implement the hypergeometric model to assess whether the number of selected genes in the ASD associated with interested items of biology knowledge is more significant than that which can be expected purely by chance. In particular, the P-value determines whether any term annotates a specified list of genes at a frequency more significant than that which can be expected by chance, as determined by the hypergeometric distribution:

$$p = 1 - \sum_{i=0}^{k-1} \frac{\binom{N-M}{n-i}\binom{M}{i}}{\binom{N}{n}}, \tag{S1}$$

where N is the total number of genes in the background distribution, M is the number of genes within that distribution that are annotated (either directly or indirectly) to the node of interest, n is the size of the list of genes of interest, and k is the number of genes within that list, which are annotated to the node. The background distribution by default is all the genes that have an annotation. Then, the outcome of a statistical hypothesis test for the hypergeometric distribution in the gene enrichment analysis, divided by the total number N of genes in the analysis, defines the enrichment efficiency η:

$$\eta = -\log_{10} P/N. \tag{S2}$$

Progressive enrichment analysis in Reactome

We make a progressive enrichment analysis from a database of Reactome to investigate the items related to DEGs. Synaptic plasticity is a crucial endophenotype in ASD, as evidenced by transcriptome enrichment of single-cell sequence data, which former genetic studies have also discovered through genetic risk genes of ASD.

The DEGs are applied to GEA in the Reactome database. At this time, we adopt a novel strategy to continuously execute the GEA procedure to find the most significant enrichment items about these DEGs across various neuro-types in ASD. As shown in Fig. 1, we noticed that the Neuronal System and the Chemical Synapses are related to DEGs. After investigating the subtype of these two parent items, we find that the NMDA and corresponding postsynaptic events (Fig. 1B) are more likely (ES=23.6% and -log10 P=14.8) to explain the dysregulation of ASD gene expression. Further inquiries into this NMDA-related item, we identify Post NMDA receptor (ES=25.0% and -log10 P=14.0) and Glutamate binding (ES=45.4% and -log10 P=10.3) in Fig. 1C.

Enrichment analysis in KEGG

Besides the Reactome database, we perform KEGG enrichment analysis for ASD DE mRNAs, considering the various biological processes. GEAs in Signal Transduction (Fig. 2) and Neuronal System (Fig. 3) items of KEGG databases illustrate that synapse and its plasticity may be the main feature in several ASD cell types.

The relationship between cerebral cortex layers and glutamate neurons

The cerebral cortex is the brain's outer layer responsible for many of the brain's higher functions, such as perception, cognition, and movement. The cortex is divided into six main layers numbered from the brain's surface (layer 1) to the border with the underlying white matter (layer 6). Glutamate is the primary excitatory neurotransmitter in the brain and plays a crucial role in many cortical processes, including synaptic plasticity, learning, and memory. Glutamate neurons are a subtype of cortical neurons that use glutamate as their primary neurotransmitter. These cells are found throughout the cortex, and their distribution varies across cortical layers. The relationship between cortical layers and glutamate

neurons is complex and varies depending on the specific region of the cortex. In general, however, glutamate neurons tend to be more abundant in the deeper layers of the cortex (layers 4-6) compared to the more superficial layers (layers 1 -3). The distribution of glutamate neurons across cortical layers reflects the functional specialization of different cortical regions. For example, cortex regions involved in sensory processing tend to have a higher density of glutamate neurons in the middle layers (layer 4). In comparison, regions involved in motor function tend to have a higher density of glutamate neurons in the deeper layers (layers 5-6). The relationship between cortical layers and glutamate neurons reflects the cerebral cortex's complex organization and functional specialization.

The relationship between ASD and intermediate neurons

Autism Spectrum Disorder (ASD) is a neurodevelopmental disorder that affects communication, social interaction, and behavior. Intermediate neurons (INs), also known as interneurons, are a type of neuron found in the central nervous system that helps to integrate and modulate signals between other neurons. While research on ASD is ongoing, there is currently no evidence to suggest a direct relationship between ASD and INs. However, studies have shown that alterations in the development and function of certain types of neurons, including INs, may be involved in the pathogenesis of ASD. For example, some studies have suggested that reduced numbers or altered functioning of certain types of interneurons may contribute to the cognitive and behavioral deficits observed in individuals with ASD. Other studies have suggested that alterations in the development and connectivity of excitatory neurons may also play a role in the development of ASD. Overall, while there is no direct relationship between ASD and INs, ongoing research is helping to shed light on the complex neurobiological underpinnings of this disorder.

INs with LTP or LTD

INs, like other neurons in the central nervous system, have glutamate receptors such as AMPA and NMDA receptors. These receptors are crucial in mediating synaptic transmission and plasticity in the brain. Additionally, studies have shown that INs can exhibit long-term potentiation (LTP) and long-term depression (LTD), which are forms of synaptic plasticity that underlie learning and memory. For example, research has shown that LTP can be induced in hippocampal interneurons, a subtype of IN, in response to high-frequency stimulation. Similarly, LTD can also be induced in interneurons, leading to decreased synaptic strength. Overall, the ability of INs to express both AMPA and NMDA receptors and exhibit synaptic plasticity suggests that they play an essential role in modulating the activity of other neurons in the central nervous system and contributing to various forms of learning and memory.

Supplementary Note S2 The Pipeline of Constructing the mSiReN

Initial nodes and final nodes in mSiReN

DEGs connected with translation control of synaptic plasticity in ASD

Among the molecules listed, the part most strongly connected with glutaminergic neurons and ASD is the glutamate receptor family, which includes AMPA receptors, NMDA receptors, and metabotropic glutamate receptors (mGluRs). Glutamate is the primary excitatory neurotransmitter in the central nervous system, and abnormalities in glutamate signaling have been implicated in ASD.

Specifically, dysregulation or dysfunction of glutamate receptors, particularly the NMDA receptor subtype, has been associated with ASD. NMDA receptors are involved in synaptic plasticity, learning, and memory, and their proper functioning is crucial for normal brain development and function. Alterations in the expression or activity of NMDA receptors have been observed in individuals with ASD, suggesting a link between glutamate receptor dysfunction and the pathophysiology of ASD.

Furthermore, metabotropic glutamate receptors (mGluRs) play a role in modulating synaptic transmission and plasticity, and aberrant mGluR signaling has also been implicated in ASD. Certain genetic variations affecting mGluR signaling have been associated with an increased risk of ASD. Some proteins or protein complexes known to be involved in synaptic plasticity, long-term potentiation (LTP), and long-term depression (LTD) exhibit specific characteristics: All mRNA precursors of AMPA, which serve as LTP, named GluAs, are upregulated. All the ARC precursors that serve as LTD, labeled as ARC

or ARP, are downregulated. The expressions of these DEGs suggest that ASD may be related to LTP, leading to the dysfunction of the translation signal network. For proteins or protein complexes closely associated with the translation control of synaptic plasticity, their expression characteristics are illustrated in a volcanic map, as shown in Supplementary Figure 6.

I. Multiple factors regulate the PIK3 pathway. It can be activated through TRK receptors (TRK->PIK3CA/PIK3CB) and G protein-coupled receptors (GPCR, specifically mGlu-> PIK3CG). Additionally, the complex "PIK3CA_PIK3R1" is regulated by the IGF pathway (IGF1R) and the RAS pathway (HRAS/NRAS/KRAS). These regulatory relationships highlight the intricate control mechanisms governing the activation of the PIK3 pathway.

II. The names Class IA and Class IB are based on the PI3K catalytic subunits. Humans express three classes of PI3K catalytic subunits, whereas Class IA specifically refers to the I-class catalytic subunits. Mammals express four Class IA catalytic subunits (p110α, β, δ and γ) encoded by PIK3CA, PIK3CB, PIK3CD, and PIK3CG, respectively. They can all phosphorylate PIP2 into PIP3. The expression of p110α and p110β proteins is widespread, while p110γ and p110β expression is enriched in immune cells. Among them, P110α, β, δ (PIK3CA, PIK3CB, PIK3CD) belong to Class IA, and p110γ (PIK3CG) belongs to Class IB. Therefore, studying Class A/B in the PI3K pathway is sufficient.

III. Metabotropic glutamate receptors (mGlu receptors) are a class of G protein-coupled receptors that play important roles in modulating synaptic transmission and neuronal activity. The composition of mGlu receptors includes the following subtypes: K04603 (GRM1), K04605 (GRM2), K04606 (GRM3), K04607 (GRM4), K04604 (GRM5), K04608 (GRM6), K04609 (GRM7), and K04610 (GRM8).

IV. "iGlu":Ionotropic glutamate receptors are a class of ligand-gated ion channels that mediate most fast excitatory synaptic transmission in the central nervous system. The composition of ionotropic glutamate receptors includes the following subtypes: K05197 (GRIA1), K05198 (GRIA2), K05199 (GRIA3), K05200 (GRIA4), K05201 (GRIK1), K05202 (GRIK2), K05203 (GRIK3), K05204 (GRIK4), K05205 (GRIK5), K05206 (GRID1), K05207 (GRID2), K05208 (GRIN1), K05209 (GRIN2A), K05210 (GRIN2B), K05211 (GRIN2C), K05212 (GRIN2D), K05213 (GRIN3A), and K05214 (GRIN3B). Specifically, GRIA1-4 and GRIN1, GRIN2A-D, and GRIN3A-B play a crucial role in synaptic transmission and neuronal signaling. The interaction between GRIN1 and GRIN2A/B/C/D forms the complex "GRIN1_GRIN2A/B/C/D," which mediates glutamate neurotransmission.

Match with annotation database

The steps to match original nodes PIK3, RAS, CAMK2, and the final node EIF4E to databases are as follows:

- To find the interesting source nodes ("receptors and upstream signals"): PIK3R1, PIK3CG, PIK3CA, HRAS, NRAS, KRAS, RASA1, RRAS, and CAMK2A, and the target nodes ("translation control"): EIF4E and EIF4EBP1.
- Match the molecules in the post-translational modified network of the Omnipath database ("named import_post_translational_interactions") and search for pathways. Complexes are not necessary for selecting initial nodes. Although complexes are present in the initial signals, they do not need to be selected because only the initial signal nodes are chosen. The signals match the corresponding downstream nodes, encompassing complexes in the interaction network, which already contain information about these complexes.

Pathway annotation databases help determine which proteins and genes are involved in other exciting pathways within the network. If the nodes in the collected signal regulatory network match in the SignaLink pathway and SIGNOR databases, we determine which genes correspond to the interested proteins and which genes do not participate in the signaling pathways. In the signal network obtained through literature mining, there are 44 "initial nodes", but after filtering by the signal network annotation, only 12 nodes remain. The molecular interaction databases match the latter to find regulatory relationships (see Table 4).

We list nodes discarded and retained by the network during the matching process with a subproject of Omnipath interaction database, named "import_post_translational_interactions". For detailed information, see Table 5.

With some super nodes not in the annotation network, match the interaction database again. Finally, In addition to the neurotransmitter receptors, three super nodes cannot match any items in the interaction database. See Table 6.

Match with interaction database

We proceed to match the molecular interaction databases. The databases "omnipath_interactions" and "pathwayextra_interactions" provide over 40,000 molecular interaction records and their intersection yields over 10,000 records. However, when searching for a maximum of eight pathways, there are over 1,000,000 records. To handle this, we extract the nodes directly from the database network, match them with the nodes from the literature-mined network, and then use the initial and terminal nodes of interest for further matching. This approach may result in a limited number of matched regulatory relationships or missing links since only a fraction of the thousands of regulatory relationships are considered when extracting over 100 nodes. Nevertheless, it remains a feasible solution.

If we were to match all nodes in the database initially and then search for subnetworks within the obtained global network, it would require significant computational r esources. Moreover, many molecular protein nodes would appear between the designated initial and final nodes in the signal network pathways, making further research challenging (additionally, after matching manually collected network nodes, there are still over 600,000 nodes).

After matching the nodes from the literature-mined network with the "omnipath_interactions" and "pathwayextra_interactions" databases, we obtain a network of 159 relationships. Following analysis and discarding nodes based on specific c onditions ("curation_effort">=2 an d "consensus_direction"= 1), we find that the constructed mRNA and protein regulatory network lacks translation control proteins (FMRP and CYFIP1), neurotransmitter receptors (mGluR and NMDAR), and regulons (ADNP, EN2, P2A, NF1, and AMPA/STK11).

Supplementary Note S3 The NIVaCaR Algorithm

We have constructed a general RNA network of protein (or complexes) precursors, mSiReN, which was further analyzed using the NIVaCaR algorithm. NIVaCaR has revealed the activity subnetworks for every cell type and allowed the identification of the core downstream modular components.

Procedure Within Mathematical Representation

Assuming a regulatory network G defined as a set of interactions $i = 1,\ldots, n_r$ and a set of species (*i.e.,* nodes) j =1,...,n_s. Each interaction i is an ordered pair of species of the form $S_i \rightarrow T^i$, where $S_i, T^i \in$ {1,...,n_s} are the source and target species, respectively. For symbol details, see Table 7. Moreover, the sign of i is denoted with $\sigma_i \in$ {-1,1}, distinguishing between activations (σ_i = 1) and inhibitions (σ_i = -1). We also define a set of cell types k = 1,..., n_e, where in each cell type a set of species are perturbed $I_{j,k} \in$ {-1,0,1} and a set of species are measured $c_{j,k} \in$ {-1,0,1}. Variables $x_{j,k} \in$ {-1,0,1} are introduced to denote the predicted activation state of species j in cell type k.

We introduce variables $u^+_{i,k} \in$ {0,1} and $u^-_{i,k} \in$ {0,1}; i = 1,...,n_r; k =1,...,n_e to denote the activity of interaction i in cell type k. The activation state of an interaction i is defined by the activity of its source node x_{S_i} and the interaction sign σ_i. The interaction has the potential to activate its target node (when $u^+_i = 1$), if and only if $\sigma_i \cdot x_{S_i} = 1$. This activation occurs in two cases: either the source node is activated ($x_j = S_i = 1$). It has an activating effect on its target node ($\sigma_i = 1$), or the source node is inhibited ($x_j = S_i$ = -1) and has an inhibiting effect (σ_i= -1). Vice versa, an interaction has the potential to downregulate its target node ($u^-_i = 1$), if and only if $\sigma_i \cdot x_{S_i}$ = -1.

For a series of cell types k, if $u_+ = 1$ then interaction i is active and can potentially upregulate its target node; else if $u_- = 1$ then interaction i is active and can potentially down-regulate its target node. An interaction i: $S_i \rightarrow T^i$ is active and may up-regulate T^i ($x^+_{i,k}$ =1), if $x_{j,k} = 1$ and $\sigma_i = 1$ or $x_{j,k}$ = -1 and σ_i = -1; $j = S_i$. On the other hand, an interaction i: $S_i \rightarrow T^i$ is active and may down-regulate T^i ($x^-_{i,k}$ =1), if $x_{j,k} = 1$ and σ_i = -1 or $x_{j,k}$ = -1 and $\sigma_i = 1$; $j = S_i$.

The rules of interactions discussed above are modeled as linear equality or inequality constraints as follows:

$$u_{i,k}^{+} \geq \sigma_i x_{j,k}; i \in \{1,\ldots,n_{\mathrm{r}}\}; j = S_i; k = 1,\ldots,n_{\mathrm{e}},$$
$$u_{i,k}^{-} \geq -\sigma_i x_j, k; i \in \{1,\ldots,n_{\mathrm{r}}\}; j = S_i; k = 1,\ldots,n_{\mathrm{e}}, \tag{S3a}$$

$$u_{j,k}^{+} \leqslant 1 - u_{j,k}^{-};\ i \in \{1,\ldots,n_r\};\ k = 1,\ldots,n_e, \tag{S3b}$$

$$u_{j,k}^{+} \leqslant \sigma_i x_{j,k} + u_{j,k}^{-};\ i \in \{1,\ldots,n_r\};\ j = S_i;\ k = 1,\ldots,n_e,$$
$$u_{j,k}^{-} \leqslant -\sigma_i x_{j,k} + u_{j,k}^{+};\ i \in \{1,\ldots,n_r\};\ j = S_i;\ k = 1,\ldots,n_e. \tag{S3c}$$

Moreover, The variables $x_{j,k}^{+} \in \{0,1\}$ and $x_{j,k}^{-} \in \{0,1\}$ denote the potential of node j being up (or down) regulated. Node j may be up-regulated ($x_{j,k}^{+} = 1$) if $\exists i$: $x_{i,k}^{+} = 1$ or $I_{j,k} = 1$. On the other hand, a node may be down-regulated ($x_{j,k}^{-} = 1$) if $\exists$ i: $x_{i,k}^{-} = 1$ or $I_{j,k} =$ -1. The activation state that node j ultimately assumes ($x_{j,k}$) is the sum of x^{+} and x^{-}. Thus, if $x_{j,k}^{+} = 1$ and $x_{j,k}^{-} = 0$, then $x_{j,k} = 1$, else if $x_{j,k}^{+} = 0$ and $x_{j,k}^{-} = 1$, then $x_{j,k} =$ -1, else if $x_{j,k}^{+} = 1$ and $x_{j,k}^{-} = 1$, then $x_{j,k} = 1$, else $x_{j,k} = 0$.

The variables are determined during the linear programming optimization according to the following set of constraints of the causal reasoning principle,

$$x_{j,k}^{+} \leq \sum_{i:T_i=j} u_{i,k}^{+}; i \in \{1,\ldots,n_{\mathrm{r}}\}; k = 1,\ldots,n_{\mathrm{e}},$$
$$x_{j,k}^{-} \leq \sum_{i:T_i=j} u_{i,k}^{-}; i \in \{1,\ldots,n_{\mathrm{r}}\}; k = 1,\ldots,n_{\mathrm{e}}, \tag{S4}$$

$$x_{j,k} = x_{j,k}^{+} - x_{j,k}^{-} + I_{j,k};\ j \in \{1,\ldots,n_s\};\ k = 1,\ldots,n_e. \tag{S5}$$

For the definition of the activation state of a node $x_{j,k}$, two cases can be distinguished: For the noninput nodes, the activity of these nodes is defined by the potentials of incoming reactions. If and only if at least one incoming reaction has the potential to activate ($u_i^{+} : T_{i=j} = 1$), the node can have the potential to be activated ($x_j^{+} = 0 \vee 1$). Vice versa, a node can only have the potential to be down-regulated ($x_j^{-} = 0 \vee 1$) if at least one incoming reaction has the potential to be inhibited ($u_i^{-} : T_{i=j} = 1$). A node is then upregulated ($x_{j,k} = 1$) if there is exclusively a potential to be upregulated ($x_j^{+} = 1$). It is down-regulated ($x_j = -1$) if there is exclusively a potential to be downregulated ($x_j^{-} = 1$). The node remains neutral if none or both potentials exist ($x_j = 0$).

Removal of feedback loops from the signaling network

In addition, feedback loops are removed given that effects mediated through those are highly dynamic and hardly interpretable from a static snapshot as in an interaction network. For instance, positive feedback loops can lead to internal signals independent of external perturbations and break the inference of pathway activities. These are constrained through a distance variable $d_{j,k}$. The distance of all nodes connected to a perturbation node is set to a value larger than zero, while all others are defined to be zero.

For example, for node j to be active ($x_{j,k} = 1$), it either has to be directly perturbed $I_{j,k} = 1$, or be activated by an upstream interaction i, such that $j = T^i$ and $x_{i,k}^{+}$ =1. However, if n nodes form a positive cycle (a cycle where all interactions are positive). One node can activate the next around the cycle without needing an external perturbation (or an incoming interaction transitively connected to a perturbation). As a consequence, only nodes connected to a perturbation can be deregulated. The distance increases from the source node to the target node if the interaction is active, *i.e.*, $u_i^{+} = 1 \vee u_i^{-} = 1$, and is not allowed to pass the distance threshold M, which is considerably larger than the expected path lengths.

The variables $d_{j,k} \geq 0$ represent the distance of node j from a perturbed node in cell type k. If node j is not connected to a perturbed node, then $d_{j,k} = 0$, else $d_{j,k} > 0$. For node j to be active, $d_{j,k} >$ 0 has to hold true. If $d_{j,k} = 0$, then $x_{j,k} = 0$. The distance of node j must be greater than that of all its upstream nodes by at least one, enforcing that the distance increases the further away we move from the input nodes, unless the upstream interactions are inactive ($i.e. u_{i,k}^{+} = x_{i,k}^{-}$ =0). Finally, the distance of any given node cannot be greater than the total number of interactions M in the signaling network. The above may be formulated using linear constraints in the following manner:

$$x_{j,k}^{+} \leqslant d_j, \quad x_{j,k}^{-} \leqslant d_j, \tag{S6a}$$

$$d_{Ti} \geq d_{Si} + 1 - M + x_{i,k}^{+}\mathring{u}M, \quad d_{Ti} \geq d_{Si} + 1 - M + x_{i,k}^{-}\mathring{u}M, \tag{S6b}$$

$$d_j \leqslant M. \tag{S6c}$$

The above constraints prohibit the ILP algorithm from conserving a positive feedback loop in the solution and allowing all the included interactions to be active unless there is an input node in the loop. Assuming that a loop like that is conserved, then the distance $d_{j,k}$ increases indefinitely in the loop, making the ILP infeasible since $d_{j,k}$ is bound by M, where M is a sufficiently significant number.

Using FC value of DEGs to discover the common core module by NIVaCaR

In the next step, we aim to apply NIVaCaR to the translated mRNA interaction network derived from the protein signaling network. We want to use the DEGs as nodes to represent the activation or inhibition status of these gene nodes in ASD relative to normal samples. We use the fold change (FC) values from Differential Expression Analysis (DEA) to determine this relative change. Using mSiReN as the prior knowledge network, we apply an integer linear programming model fitting using the differential expression values of the gene nodes. This model helps to identify which nodes and pathways are activated in ASD and to investigate whether there is specificity or commonality across different types of neurons.

At the algorithmic level, we select the DEGs for each cell type that show dysregulation in ASD at the RNA expression level. These genes represent the dysregulated response in ASD at the RNA level. The FC values of these genes are binarized to determine whether they are upregulated or downregulated in ASD cells. The framework consists of the following steps:

- DE gene expression: We start with gene expression data from different scRNA-seq data. These data reflect the relative expression levels of genes across the cell types and CTL/ASD.
- Binarization: We binarize the gene expression data to simplify the analysis and focus on pathway activation. This binarization involves setting a threshold to categorize genes as either "active" or "inactive" based on their expression levels. This step transforms the continuous gene expression data into a binary representation.
- NIVaCaR algorithm: We apply the NIVaCaR algorithm, based on causal reasoning and network identification, to the binary gene expression data. NIVaCaR aims to identify the activated pathways by analyzing the relationships between genes and network structure.
- Integer Linear Programming (ILP): We utilize an ILP framework within the NIVaCaR algorithm to infer the most likely activated pathways. ILP allows us to optimize the pathway activation patterns based on the binary gene expression data.

The activated subnetworks are illustrated in Fig. 4 for different excitatory cell types and Fig. 5 for inhibitory types from NIVaCaR results. After comparing the results of NIVaCaR with the gene expression, we find that EIF4EBP is not a DEG (for example, in L4, logFC=-0.0024, AveExpr=0.0216 and P.Value=0.711894) but still follows a causal logic of an "activated signaling pathway." It indicates that NIVaCaR identifies causal relationships in network regulation processes rather than solely relying on gene expression levels to determine potential dysregulated molecules (see Fig. 11).

Supplementary Note S4 The ProComReN Algorithm

Their regulatory networks' properties largely determine eukaryotic cells' functional characteristics. Even with the vast amount of biological data accumulated over the past decades, a global model of how these networks determine the phenotypes of healthy and diseased cells remains elusive. One goal of systems biology is to understand these networks at the highest possible protein functional level, for example, to devise therapeutic strategies. Mathematical modeling of regulatory networks allows for

discovering knowledge at the systems level. However, existing modeling tools are often computation-heavy, such as ordinary differential equation (ODE) formula, and do not offer intuitive ways (logical algebraic parameter, such as CNORfuzzy [1]) to explore the model, to test hypotheses, or to interpret the results biologically.

Numerous mathematical approaches exist to optimize and train regulatory network models against steady-state experimental data. Of these, logical models [2] are of particular interest, as they can capture essential features of the system being modeled and generate biological insights while requiring less prior knowledge and experimental observations than differential equation models [3]. In addition, logical models are generally more potent than statistical models, as they incorporate the relational information embedded in the network structure. In contrast, statistical models aiming at reverse-engineering biological networks from high-throughput data implicitly consider all possible topologies [4]. Some successful applications include the logical models of yeast cell cycle protein network [5], gene regulatory networks [6], signaling networks [7].

In logical models of systems at steady-state, nodes represent the degree of activation of the system's constituents at equilibrium, and edges represent the logical functions between nodes. These functions can be either linear or nonlinear functions of the parent nodes and are combinations of the fundamental "AND", "OR", and "NOT" Boolean functions. While Binary Boolean models [8] only consider full activation or complete absence, more quantitative approaches, for instance, Probabilistic Boolean Networks (PBNs) [9] and Dynamic Bayesian Networks (DBNs) [10] can account for intermediate or continuous activation values and allow the integration of data uncertainty. Monte Carlo usually analyzes these approaches, which can be computationally demanding or non-intuitive.

Here, we have developed a computational approach, ProComReN, to efficiently contextualize logical models of regulatory networks (very suitable for signal transduction network) with biological measurements (scRNA-seq data) based on a probabilistic description (PBN) of rule-based interactions (or DBN with algebraic formula) between the different species (molecules or nodes). This algorithm is inspired by FALCON [11], Fuzzy [3], and CNOprob [12].

The tool presents a computational approach to contextualize logical models of regulatory networks designed explicitly for signal transduction networks. It facilitates the integration of biological measurements, such as cell-specific scRNA-seq data, into the modeling process. The approach is based on a probabilistic description, utilizing either Probabilistic Boolean Networks (PBN) or Dynamic Bayesian Networks (DBN) with algebraic formulas to capture the rule-based interactions between the different species or nodes within the network.

A Bayesian interpretation of the logical "gates" allows for an algebraic formulation of the system and an efficient calculation of the long-term steady-state of the system given the specified inputs. A gradient-descent optimizer minimizes the error function calculated as the sum of squared residuals between the simulated and experimentally measured nodes of interest. This approach can encompass a broader range of potential regulatory edges without delving into the intricate molecular mechanisms underlying each interaction. This strategy effectively reduces the model parameters, enabling increased computational efficiency, providing possibilities for training large-scale biological networks, and facilitating the post hoc analyses detailedly.

It is important to note that the model operates on synchronized update data, as the signal transduction network directly regulates the translation control network. Subsequently, the model maps the translated information onto its mSiReN for analysis. Asynchronous updates are not considered in this context, and the logical formulation of the interaction between different molecules is intuitive. ProComReN is an algorithm for the efficient contextualization of logical network models, which provides essential qualitative and quantitative information about the system being modeled. Specifically, ProComReN is well suited to assess the relative contributions of different signal regulatory mechanisms to the system's behavior at steady-state.

The mathematical representation of the ProComReN algorithm

There is a clear conceptual difference between differential equations and coarse-scale models. The former can be used for a detailed representation of biochemical reactions, whereas the latter emphasizes fundamental, generic principles between interacting components. In this context, the theory of computational models [10] classes that both discrete-time and discrete-state are called coarse-scale models.

Limitations of differential equations for fine-scale modeling of biological interactions at the molecular level are:

- Those models are computationally very demanding.
- The model selection problem is usually ignored.
- The underlying biological system is assumed to be known.

The so-called graphical models can overcome the modeling issues mentioned above problems, and advanced analysis tools have been developed for them.

The use of holistic, coarse-scale models is also supported by the fact that the currently available data is limited in quality and the number of samples. That is, there is no advantage to using models that are much more accurate than the available data. Another constraint to remember is that the modeling framework should be selected based on the preferred goals, *i.e.*, the kinds of questions we seek answers to.

The relationship between PBN and DBN

A Probabilistic Boolean Network (PBN) is a specific type of dynamic Bayesian network (DBN) used to model and analyze the behavior of complex systems, particularly in computational biology. The main idea behind PBNs is to represent the state of each variable in the system as a Boolean value (either true or false) and introduce probabilistic transitions between the states of these variables over time. Unlike traditional Boolean networks, where the transitions between states are deterministic, PBNs incorporate uncertainty by assigning probabilities to the state transitions.

In a PBN, the system is modeled as a directed acyclic graph (DAG) consisting of Boolean variables as nodes and probabilistic dependencies as edges. Each node represents a Boolean variable, and the edges represent the probabilistic influence of the parent nodes on the state of the child node. The probabilities associated with the edges define the transition probabilities between the states of the variables. The transition probabilities in PBNs can be specified in different ways. One common approach is to use a Boolean function or a logical rule to determine the probability of transitioning from one state to another. These functions can be defined based on prior knowledge, experimental data, or expert opinions. PBNs are particularly useful for modeling and analyzing biological systems, such as gene regulatory networks. They allow researchers to capture the stochastic behavior and inherent uncertainties in biological processes. By simulating the dynamics of the PBN, researchers can gain insights into the system's behavior, make predictions, and study the effects of perturbations or interventions.

The relationship between PBNs and DBNs lies in the fact that PBNs are a specific type of DBN. As mentioned earlier, DBNs are a general class of models that capture temporal dependencies and uncertainties in dynamic systems. PBNs, being a specific instance of DBNs, focus on representing Boolean variables with probabilistic transitions. Therefore, PBNs can be seen as a specialized form of DBNs tailored for modeling Boolean systems with probabilistic behavior. It is worth noting that while PBNs are widely used in computational biology, DBNs have broader applications beyond Boolean systems and can model various types of variables with different probability distributions, continuous or discrete.

Boolean functions or logical rules are crucial in specifying the transition probabilities within PBNs. In a PBN, Boolean functions or logical rules determine transition probabilities. In a PBN, each node can exist in different states, typically represented as binary values (*e.g.*, "On" or "Off", "1" or "0"). The transition probabilities describe the likelihood or probability of a node transitioning from one state to another.

Explanation for edges in ProComReN

The passage describes the meaning and constraints of the weights associated with edges and hyperedges in a modeling framework. Each edge or hyperedge in the network is assigned a weight, denoted as $k_j^{(i)}$, representing the relative influence of the upstream node to the downstream node. In this Bayesian-based modeling framework, the weights must adhere to the law of total probability. Specifically, for each node$X^{(i)}$ that has a set of m activating functions denoted as j_+, the sum of the activating weights $\sum_{j_+=1}^{m} k_{j_+}^{(i)}$ must be equal to 1. The weights associated with activating interactions should collectively

account for the total influence on the downstream node. Similarly, for nodes that have a set of l inhibiting functions denoted as j_-, the sum of the inhibiting weights $\sum_{j_-=1}^{l} k_{j_-}^{(i)}$ must be between 0 and 1. It ensures that the weights of inhibiting interactions represent the relative inhibition of upstream nodes and are within a valid range. In summary, the weights in the modeling framework follow the principles of total probability, ensuring that the activating weights sum up to 1 and the inhibiting weights fall within the range of 0 to 1.

Given a network structure established from prior knowledge, a set of parameters (weights), and a set of experimental conditions, the steady state of the network is computed for each of the conditions, and the values of the nodes corresponding to the measured species are recorded. For each of the conditions, the network nodes are initialized with random values, except for the nodes considered as inputs (external to the system), for which the value is determined by the experimental conditions and kept constant. The network is then updated repeatedly by computing synchronously for each node the expected value of its probability distribution, given the value of its parent nodes and the weights associated with each interaction.

Because all nodes at each update are considered independent, the input values of "AND" logical gates are multiplied. The computation of "OR" gates follows De Morgan's law, *i.e.*, the complement of the union of two sets is the same as the intersection of their complements. Inputs pointing to the same child node that are not members of a logical gate are summed. Table 9 summarizes the different types of interactions explicitly formulated in our framework. The algebraic formulas used for the computations can be directly derived from the conditional probability tables of the DBN formulation of the logical interactions. The resulting dynamical system converges to a steady state where each node value corresponds to the normalized equilibrium concentration of the activated form of the molecule in the system.

Assignment of additive effects to logic gates

One significant difference in the ground assumptions needs to be discussed. When more than one interaction comes to a node, the CellNOpt packages assume that these interactions either take the OR or the AND gate. Alternatively, ProComReN considers the third case, like CNOprob, *i.e.*, an additive effect without assigning the OR/AND gate in this scenario. This assignment gives more flexibility to the ProComReN framework as more types of reactions with a broader set of transfer functions can be assigned.

PBNs and Boolean functions or logical rules

The relationship between Probabilistic Boolean Networks (PBNs) and Boolean functions or logical rules lies in predicting transition probabilities within PBNs. In PBNs, the transition probabilities represent the likelihood of transitioning from one state to another in the network. These probabilities can be determined using various approaches, and one standard method is Boolean functions or logical rules. Boolean functions or logical rules formally represent the relationships and interactions among the network's variables (or nodes). These functions define the conditions under which a transition from one state to another occurs and assign probabilities accordingly. The Boolean functions can be derived from prior knowledge, experimental data, or expert opinions regarding the modeled system.

PBNs allow for a probabilistic description of the network dynamics by employing Boolean functions or logical rules. The transition probabilities are determined based on the satisfaction of the Boolean functions or logical rules, enabling the modeling of complex behaviors and capturing uncertainty in the system. In summary, Boolean functions or logical rules are crucial in specifying the transition probabilities within PBNs, facilitating the probabilistic modeling of regulatory networks, and capturing the system's dynamics.

A toy example for PBN and boolean functions or logical rules

A simple toy example illustrates the relationship between Probabilistic Boolean Networks (PBNs) and Boolean functions. Imagine a regulatory network consisting of Gene A, B, and C genes. Each gene can exist in two states: 'On' or 'Off.' The interactions between these genes govern the behavior of the network. In a PBN, we assign transition probabilities to determine the likelihood of transitioning from

one state to another. These probabilities are determined by using Boolean functions or logical rules. For our example, we assume the following Boolean functions for each gene:

- Gene A: If Gene B is 'On' and Gene C is 'Off,' the probability of Gene A transitioning from 'Off' to 'On' is 0.8. Otherwise, the probability is 0.2.
- Gene B: If Gene A is 'On,' the probability of Gene B transitioning from 'Off' to 'On' is 0.6. Otherwise, the probability is 0.4.
- Gene C: If Gene A is 'On' and Gene B is 'On,' the probability of Gene C transitioning from 'Off' to 'On' is 0.9. Otherwise, the probability is 0.1.

These Boolean functions specify the conditions under which a transition occurs and assign corresponding probabilities. They are derived from prior knowledge, experimental data, or expert opinions about the regulatory interactions between the genes. Using these Boolean functions, we construct the transition probabilities for the PBN. These probabilities capture the dynamics of the network and allow for probabilistic reasoning about the system's behavior. For instance, based on the Boolean functions, if Gene A is currently 'Off' and Gene B is 'On,' the probability of Gene A transitioning to 'On' in the next time step is 0.2. Similarly, the probabilities of Gene B and Gene C transitioning between states are calculated based on their respective Boolean functions and the current states of the genes. By simulating the PBN over time or performing probabilistic analyses, we gain insights into the behavior of the regulatory network and the probabilities associated with different gene states. In summary, in a PBN, Boolean functions or logical rules are used to determine transition probabilities, which describe the likelihood of transitioning between states in the network. These functions capture the regulatory interactions and are used to model and analyze the behavior of the network in a probabilistic manner.

Transitioning from one state to another

"State Transition" in the context of PBNs refers to the change in the state of a node or variable within the network. Each node in a PBN can exist in different states, typically represented as binary values ('On' or 'Off,' '1' or '0'). The transition probabilities describe the likelihood or probability of a node transitioning from one state to another. For example, consider a gene in a regulatory network represented by a PBN. The gene can be in an 'On' or 'Off' state. The transition probabilities associated with this gene would indicate the likelihood of it transitioning from an 'Off' state to an 'On' state or vice versa. These transition probabilities capture the network dynamics and provide information about the probability of a node changing its state based on the current states of its input nodes or other factors. They reflect the influence of regulatory interactions and external factors on the behavior of the network. By specifying and analyzing these transition probabilities, we can understand how the network evolves and make probabilistic predictions about the states of the nodes in the network at different time points.

The segments of ProComReN algorithm

Objective function

We employ a steady-state analysis approach to contextualize the model with experimental data. We extract the values of nodes in the network that correspond to the measured data and compare them with the normalized values obtained from experimental observations. The mean squared error (MSE) is then computed to quantify the discrepancy between the estimated values and the actual measurements. We utilize a gradient-descent algorithm to optimize the weights and minimize this error measure. To ensure computational efficiency while accommodating varying degrees of recurrence in the networks, we employ the interior-point method [13]. This method allows us to strike a balance between accuracy and computational feasibility.

Differential regulation

In many real-life modeling applications, a system is studied in different contexts. For example, the same signaling pathways are studied for different cell lines or over time during a drug screen. One goal of systems biology is to identify differences between the contexts in regulating the system. The same prior

knowledge model is contextualized in parallel with different datasets corresponding to different contexts. ProComReN automates such analyses by optimizing identical models in parallel for multiple series of experimental conditions (cell types). We can discover which network parts are activated or shut down between cell lines/time points, which may lead to identifying specific intervention strategies for each context.

Rapid optimization

Using the gradient-descent optimization algorithm fmincon (find a minimum of a constrained nonlinear multivariable function) with interior-point method [14, 15], ProComReN can rapidly estimate the set of weights that minimizes the objective function. Random initialization of the weights is done either from a uniform distribution across the [0, 1] range or from a truncated normal distribution centered on 0.5.

Supplementary Note S5 The Pipeline of mSiReN Reduction for ProComReN Algorithm

The Criterion of filtering nodes in mSiReN:

1. FC value (P-value)
2. Expression is not too low
3. Topology of the network
4. Priori knowledge

To determine the checkpoint nodes in the upstream, two criteria need to be met: Criterion 1, which applies to both some other nodes and to upstream nodes, and Criterion 2, which applies only to the other nodes. However, the upstream nodes should meet Criterion 2 but not Criterion 1. It is because the control or ASD states refer to the entire system, including all nodes and edges. In contrast, the upstream nodes serve as a classification reference for other nodes, providing a binary criterion based on their expression. On the other hand, the downstream nodes should not meet criterion 2.

To understand the origin of these downstream components, especially one core module containing EIF4EBP1 and EIF4E, we perform the reverse tracing method, which constantly looks for input nodes to determine the upstream regulatory network. Not only do these nodes need to pass the filter of 7 nodes and ten edges in the inverse trace, see Fig. 12, but there are many nodes that are crucial for the network but exhibit lower expression.

The significant difference between the method of the union network derived from the NIVaCaR results of cell types and the core module-based inverse trace network (Fig. 13) lies in the expression or fold change (FC) value of nodes in the network. In the former approach, NIVaCaR utilizes the FC values to filter and label nodes as negative or active, executing the algorithm to identify logically activated pathways or subnetworks. However, in the latter approach, nodes in the entire network are not selected based on FC values, and the ProComReN algorithm remains valid even if some nodes are not differentially expressed genes (DEGs) in the network. Therefore, if the ProComReN method is to be used, the original network should be employed instead of the network derived from the NIVaCaR results, which include filtered nodes based on FC values.

If nodes with expression lower than 20% in the specific cell type are deleted, the node EIF4EBP would be removed (Fig. 14). This general expression filter applied to the "translation control" part results in the deletion of EIF4EBP. However, the ProComReN analysis still handles this node appropriately. Despite its low expression, EIF4EBP can be included in the analysis. Its input edges may be less activated in the network. The analysis can proceed even with one or two nodes exhibiting low expression levels.

The network represents a regulatory system with multiple nodes interconnected by edges. The upstream nodes, including CAMK2A, HRAS, KRAS, NF1, PIK3CA, PPP2CB, PRKAA1, PRKAA2, PTEN, RAC1, STK11, and SYNGAP1, play crucial roles in influencing the downstream behavior of the network. CAMK2A and SYNGAP1 are isolated among these upstream nodes, indicating that they do not receive direct inputs from other nodes within the network. This isolation suggests their regulatory effects may be unique or independent of other nodes.

Additionally, the network (Fig. 15) contains nodes such as MAP3K7 and MAP3K2, which are not considered part of the upstream nodes but still serve as input nodes based on the network's topology. These nodes may contribute to the overall behavior of the network, although they are not directly involved in the regulation of the upstream nodes. Specific nodes in the network, namely HRAS, PIK3CA, RAC1, PRKAA1, and PRKAA2, have incoming edges deemed essential and should not be deleted. These edges likely represent significant regulatory interactions that are crucial for the proper functioning of the network. Nevertheless, based on the input nodes' selecting rule: "Target more upstream pathway", Discard PPP2CB and PTEN.

Finally, we noticed some nodes have homologs such as AKT2, MAP2K1, and PRKAA1 (see Table 8), but it has lower expression in ASD. After filtering homolog with lower expression, AKT2, MAP2K2, and PRKAA1 (Fig. 16), we obtain the final network structure (Fig. 17), which have 25 nodes and 43 edges containing RHEB, TSC2, RAF1, EIF4EBP1, MTOR, MAP2K1, NF1, MAPK1, MAP2K2, HRAS, KRAS, PTEN, MKNK1, PDPK1, AKT3, TSC1, PRKAA1, RPS6KA5, AKT2, GSK3B, PRKAA2, STK11, PIK3CA, RAC1, PPP2CB, RPTOR, RPS6KB1, and EIF4E.

Supplementary Note S6 Contextualizing mSiReN with ProComReN

To gain a deeper understanding of the underlying regulatory mechanisms, we employ a boolean logic model, which allows us to contextualize the gene expression data into a quantitative model. In order to capture the cell-specific behavior, we employ CS-ProComReN to create cell-specific quantitative models. This model incorporates the regulatory probabilities specific to each cell type, providing insights into the dynamics and behavior of the system at a cellular level. Hence, we establish a pipeline to construct CS-ProComReN from a PKN and perturbation expression (inspired by the method of discrete logic modeling [7]), which integrates scRNA-seq data and puts the regulation network into a boolean logic model.

Establishing the experimental data-based quantitative model

Our capacity to generate large datasets is increasing steadily. A helpful way to extract mechanistic insight from the data is by integrating them with a prior knowledge network of signaling to obtain dynamic models. Logic networks are among the conceptually most straightforward modeling frameworks. They capture the mechanistic relationship between molecular entities. Due to their simplicity, they are highly scalable and widely applied. The molecular changes induced by perturbations such as drugs and ligands are highly informative of the intracellular wiring. For the detailed and coherent experimental data-based quantitative models, we directly contextualize mSiReN into scRNA-seq information.

Experiment-liked data to train the model

We classify a specific node's high or low expression in all cells belonging to one neuron type to set the perturbation experimental data. Based on this node's (also gene) expression, we continue to add another perturbation node. Those combinations of perturbation expression of nodes are similar to the data from the cell line experiment.

We employ a combination of Genetic Algorithm (GA) and Integer Linear Programming (ILP) techniques to optimize the model parameters and infer the regulatory relationships. This optimization approach enables us to refine the model and uncover the critical regulatory elements associated with ASD. By applying the GA/ILP-based optimization, we identify the specific regulatory mechanisms that distinguish ASD from control samples. This approach provides valuable insights into the dysregulated pathways and potential molecular targets underlying ASD pathogenesis.

The preprocessing of data for the ProComReN model

Linear function normalization is a technique used to transform raw data into a range of [0-1], simplifying downstream analysis while preserving the relationships present in the original data. This method assumes that the maximum and minimum values across the samples are similar. The formula for linear function normalization is

$$\frac{x - \min(x)}{\max(x) - \min(x)}, \tag{S7}$$

where x represents the data points.

Use the scRNA-seq expression data of CTL and ASD as the input of modeling

The proposed approach integrates the original mSiReN with single-cell RNA sequencing (scRNA-seq) expression data to comprehensively analyze pathway networks. For detail, the mSiReN captures the intricate regulatory relationships among precursor mRNAs of proteins. At the same time, scRNA-seq expression data provides high-resolution information on gene expression patterns at the single-cell level. This network structure and information combination allows for a deeper understanding of the underlying regulatory mechanisms governing signal transduction networks, facilitating the identification of critical regulators and elucidating complex translation control processes in ASD. Therefore, we use the original whole mSiReN from the interaction database through a manual signal transduction network to execute the ProComReN directly to establish a quantitative model. discrepancy

Assessing discrepancies between two main cell types

Assessing the discrepancy of excitatory cell types (L, L23, L4, L56, and L56CC) and inhibitory cell types (IN, INPV, INSST, INSV2C, and INVIP) ensures network stability after applying an expression-based filter to the PKNs of various cell types for ProComReN. We calculate the variation of the Standard Error (SE) of expression for L and IN cell types (refer to **Supplementary File 8**). The results indicate that L and IN represent distinct majority cell types based on the expression of the nodes of interest. Consequently, we separate these two main cell types for further analysis. On the other hand, more than 100 cells of input nodes remain to be calculated in subsequent steps after filtering out the non-expression part, as depicted in Fig. 21. Input nodes in the primary cell type "Neu" ("Neumat", "NeuNRGNI", and "NeuNRGNII") exhibit the highest degree of zero expression. Deleting these cells leads to an unstable outcome during the training of ProComReN, as illustrated in Fig. 22.

Supplementary Note S7 Relationship between NIVaCaR and ProComReN

General network structure and specific modeling

The constructed signal network is from literature and databases; each cell type has the same signal network. Similarly, the network structure of mSiReN is for all cell types. However, the expressions of nodes and FC values are different for a specific cell type. Therefore, the activated subnetworks and sub-pathways are unique in the following modeling methods.

The NIVaCaR algorithm treats each cell's expression data (combining all cells within that cell type) as an independent unit. The collected and organized signal network and the signal network built using Omnipath is used as the target network for performing NIVaCaR's network activation analysis.

For ProComReN modeling analysis, a signal network is obtained from a specific type of cell, the gene node data is Booleanized (or organized in "probability form"), and a Boolean network model is built to explore the dynamics of ASD synaptic disorders under the influence of different upstream signaling molecules. NIVaCaR and ProComReN are not sequential methods but are parallel. NIVaCaR identifies the activated subnetworks in mSiReN for each cell type and discovers the core module of the EIF4E and EIF4EBP1 pair to further model in ProComReN. If these nodes in the activated subnetworks are used to construct a new network, it lacks coherence in the network structure.

The combinations of NIVaCaR and ProComReN

Next, we check whether the result of subnetworks from NIVaCaR can be uniform to find a suitable and general network for further quantitative analysis by ProComReN.

- If NIVaCaR 1, ProComReN 1, then feasibility 0: cannot find a uniform subnetwork for all cell types.
- If NIVaCaR 0, ProComReN 1, then feasibility 0: ProComReN cannot decide which jointed network to implement (46 nodes are too many).
- If NIVaCaR 1, ProComReN 0, then feasibility 1: Lose the quantitative description of ProComReN.

For the network structure to train the model, if we use the activated nodes and edges found directly by the NIVaCaR, there could not be enough consistent nodes and edges, see Fig. 18.

The problem for core module-based inverse trace

However, if we use inverse trace Fig. 12 in the network, almost all nodes are included after several neighbor incoming edges (finally discard five nodes). On the other hand, based on the "core module", the inverse trace network is also going to face the "all incoming edges" problem, such as TSC2 has all the effects of other nodes, which any cell type only cannot have. The third question is whether these edges direct from the interaction database exist in every cell type without using the RNA-seq data-based method, NIVaCaR.

The limit of union network to train the model

We might need to use the unique or common network Fig. 19 and Fig. 20 from the results of all cell types and make further quantitative analyses by ProComReN. The conception of upstream nodes and the transmitter receptor is used to NIVaCaR before the ProComReN pipeline. Moreover, in the process of ProComReN, we can focus more on customized subnetworks across cell types.

When employing the union network that incorporates all L or IN-type neurons, each node in the network becomes a fusion of multiple neuro-types. For instance, taking TSC2 as an example, it represents a blend of four different neuron types, each with its unique set of connections. Utilizing the ProComReN method to identify universal paths in the network based on specific cell type data may result in certain paths only relevant to that particular cell type. These paths might not exist in other cell types. Furthermore, the overall network structure, including the arrangement of edges, could vary significantly across different cell types.

In conclusion, the NIVaCaR and ProComReN methods are incompatible, making them unsuitable for direct combination. Both techniques aim to discover consistent pathways in a network that can match experimental data but differ in their underlying approaches. NIVaCaR relies on FC values, while ProComReN relies on gene expression data directly. Since the two methods differ in methodologies and data requirements, attempting to merge them directly may not yield meaningful or coherent results.

Supplementary Note S8 The Significance of mSiReN in Investigating Dysfunctions in Signal Transduction

Gene mutation, RNA, protein, and phenotypic effects

The term "endophenotype" refers to a measurable and heritable characteristic or trait intermediate between a specific genetic variation and a complex phenotype or clinical manifestation of a disorder. Endophenotypes are believed to be more closely related to the underlying genetic mechanisms of a disorder than the observable symptoms or clinical diagnosis. They are often used in research to study complex traits' genetic and biological basis, such as psychiatric disorders, by providing a more direct link to the underlying genetic factors. Endophenotypes can include physiological, biochemical, cognitive, neuroanatomical, or neurophysiological measures associated with a particular disorder. By studying endophenotypes, researchers aim to understand disorders' genetic basis and mechanisms better and improve diagnostic accuracy and treatment approaches.

A gene mutation results in the production of a faulty RNA molecule, which can ultimately lead to the production of a dysfunctional protein. This cause can occur when the mutation alters the gene sequence in a way that disrupts the normal process of transcription, during which the DNA sequence is copied into RNA. The faulty RNA molecule can then be translated into an incomplete or misfolded

protein, which can result in the protein losing its normal function. In some cases, mutations in a single gene can result in downstream effects on other genes or proteins, leading to a convergence of phenotypic effects. Examples of such conditions include cystic fibrosis, sickle cell anemia, and Huntington's disease, all caused by mutations in a single gene that produce dysfunctional proteins with downstream effects on cellular processes.

The necessity of training quantitative model

Several reasons underscore the necessity of training quantitative models for RNA levels or transcriptomics:

1. RNA serves as a precursor for protein synthesis, and the RNA levels correlate positively with protein levels within a specific time frame.

2. ASD research differs from cancer research, where abundant cell lines and wet lab experiments can collect perturbation data to observe changes in signaling networks under various external stimuli.

3. While a significant portion of research focuses on gene regulatory networks (GRN), the impact of external stimuli on GRN networks and the downstream pathways of essential genes remain uncertain.

4. ASD, a psychiatric disorder, involves the study of humans, as opposed to animal models, making it impossible to collect real-time samples for biochemical experiments to determine the molecular mechanisms in pathology.

The advantages of mSiReN in investigating dysfunction mechanisms

The mRNA Signaling-Regulatory Network (mSiReN) plays a crucial role in understanding the intricacies of signal transduction related to synaptic plasticity and translation control 9. Here are the key advantages of mSiReN in investigating dysfunction mechanisms in the signal transduction network:

- mRNA as a Determinant of Protein Expression: mRNA is a pivotal factor influencing protein expression and provides valuable insights into potential dysfunctions in protein production within the transduction network. It extends beyond protein identification, encompassing regulatory interactions from the RNA world to the functional level of proteins. This understanding sheds light on the convergence of evidence associated with ASD, such as mRNA, non-coding RNA (ncRNA), and protein interactions in specific cell types, particularly in the DLPFC (Dorsolateral Prefrontal Cortex).

- Utilization of Abundant RNA Sequencing Data: mSiReN's modeling approach relies on extensive RNA sequencing expression data. This approach allows for exploring interactions between mRNA molecules, leading to a deeper understanding of the mechanisms underlying aberrations in the signal transduction network.

- Identification of Dysregulated RNA: mSiReN identifies dysregulated protein-coding RNA (mRNA) and uncovers intricate regulatory relationships involving non-coding RNA. It is a significant contribution, as the protein-RNA interactions within the signal network, well-established in the ASD community, have not been fully explored. Our work introduces the possibility of aberrations at the RNA level, specifically RNA-ncRNA interactions, within the signal transduction network.

The comprehensive approach offered by mSiReN holds the potential to reveal novel insights into the molecular mechanisms contributing to ASD, especially concerning translation control in synaptic plasticity. Moreover, it opens avenues for the development of targeted therapeutic strategies aimed at specific endophenotypes of ASD.

References

[1] Terfve, C. *et al.* Cellnoptr: a flexible toolkit to train protein signaling networks to data using multiple logic formalisms. *BMC systems biology* **6**, 1–14 (2012).

[2] Le Novere, N. Quantitative and logic modelling of molecular and gene networks. *Nat Rev Genet* **16**, 146–58 (2015). URL `https://www.ncbi.nlm.nih.gov/pubmed/25645874`.

[3] Morris, M. K., Saez-Rodriguez, J., Clarke, D. C., Sorger, P. K. & Lauffenburger, D. A. Training signaling pathway maps to biochemical data with constrained fuzzy logic: quantitative analysis of liver cell responses to inflammatory stimuli. *PLoS Comput Biol* **7**, e1001099 (2011). URL `https://www.ncbi.nlm.nih.gov/pubmed/21408212`.

[4] Bansal, M., Belcastro, V., Ambesi-Impiombato, A. & Di Bernardo, D. How to infer gene networks from expression profiles. *Molecular systems biology* **3**, 78 (2007).

[5] Li, F., Long, T., Lu, Y., Ouyang, Q. & Tang, C. The yeast cell-cycle network is robustly designed. *Proceedings of the National Academy of Sciences* **101**, 4781–4786 (2004).

[6] Mendoza, L., Thieffry, D. & Alvarez-Buylla, E. R. Genetic control of flower morphogenesis in arabidopsis thaliana: a logical analysis. *Bioinformatics (Oxford, England)* **15**, 593–606 (1999).

[7] Saez-Rodriguez, J. *et al.* Discrete logic modelling as a means to link protein signalling networks with functional analysis of mammalian signal transduction. *Molecular Systems Biology* **5** (2009).

[8] Kauffman, S. A. Metabolic stability and epigenesis in randomly constructed genetic nets. *Journal of theoretical biology* **22**, 437–467 (1969).

[9] Trairatphisan, P. *et al.* Recent development and biomedical applications of probabilistic boolean networks. *Cell communication and signaling* **11**, 1–25 (2013).

[10] Lähdesmäki, H., Hautaniemi, S., Shmulevich, I. & Yli-Harja, O. Relationships between probabilistic boolean networks and dynamic bayesian networks as models of gene regulatory networks. *Signal processing* **86**, 814–834 (2006).

[11] De Landtsheer, S., Trairatphisan, P., Lucarelli, P. & Sauter, T. Falcon: a toolbox for the fast contextualization of logical networks. *Bioinformatics* **33**, 3431–3436 (2017). URL `https://www.ncbi.nlm.nih.gov/pubmed/28673016`.

[12] Gjerga, E. *et al.* Converting networks to predictive logic models from perturbation signalling data with cellnopt. *Bioinformatics* **36**, 4523–4524 (2020). URL `https://www.ncbi.nlm.nih.gov/pubmed/32516357`.

[13] Waltz, R. A., Morales, J. L., Nocedal, J. & Orban, D. An interior algorithm for nonlinear optimization that combines line search and trust region steps. *Mathematical programming* **107**, 391–408 (2006).

[14] Coleman, T. F. & Li, Y. An interior trust region approach for nonlinear minimization subject to bounds. *SIAM Journal on optimization* **6**, 418–445 (1996).

[15] Coleman, T. F. & Li, Y. On the convergence of interior-reflective newton methods for nonlinear minimization subject to bounds. *Mathematical programming* **67**, 189–224 (1994).

Supplementary Figure

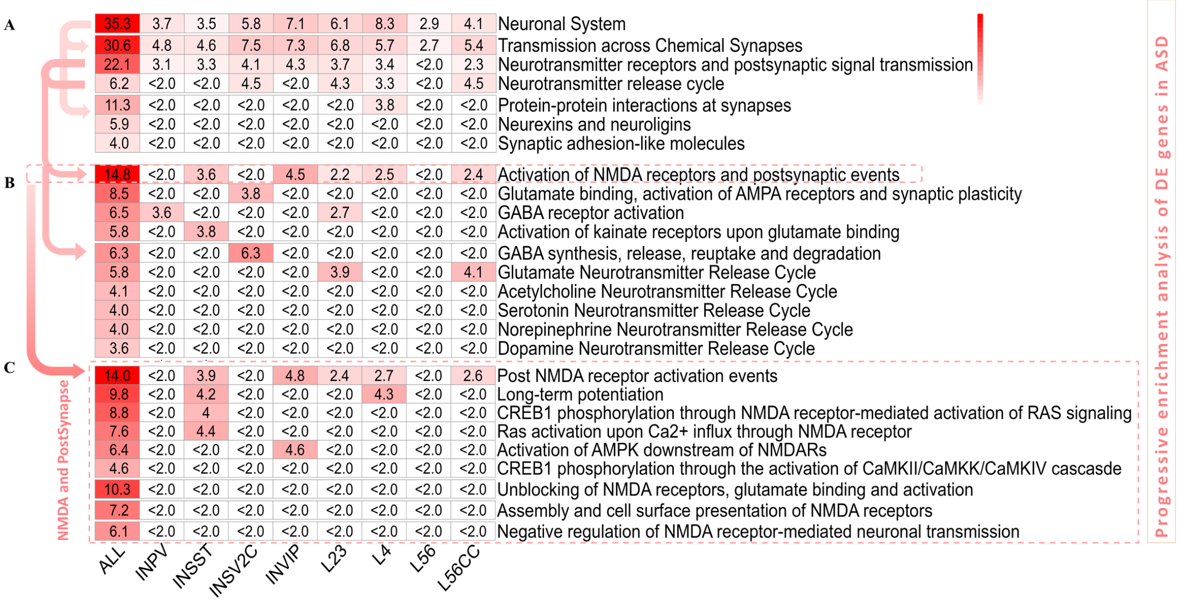

Figure S1. Synapse-related endophenotype as ASD dysfunction discovered from the evidence of enrichment analyses of DEG in ASD.

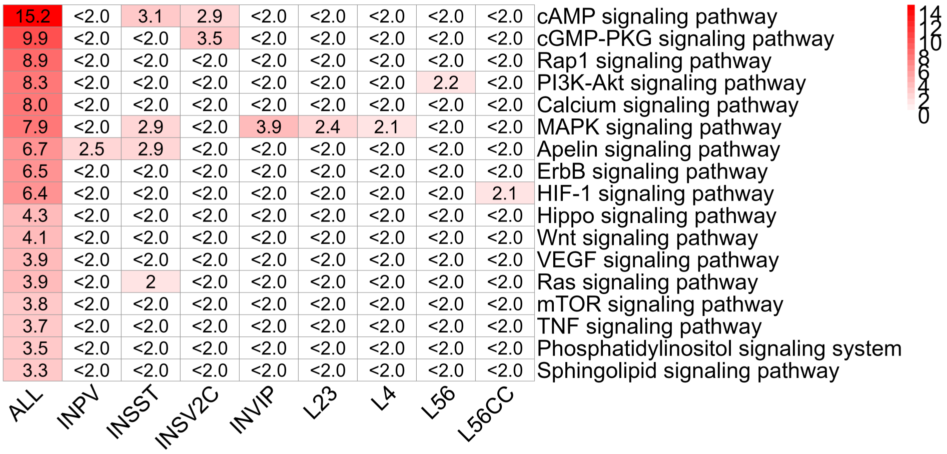

Figure S2. KEGG enrichment of DE mRNA for Signal Transduction items in 8 types of brain cells.

	ALL	INPV	INSST	INSV2C	INVIP	L23	L4	L56	L56CC
Retrograde endocannabinoid signaling	15.5	7.3	8.5	3.5	3.5	5.3	6	<2.0	2.6
Cholinergic synapse	10.9	<2.0	<2.0	<2.0	<2.0	<2.0	<2.0	<2.0	<2.0
Long-term potentiation	9.9	<2.0	<2.0	<2.0	<2.0	<2.0	<2.0	<2.0	<2.0
Synaptic vesicle cycle	9.9	<2.0	<2.0	2.6	<2.0	2.4	<2.0	<2.0	2.6
Glutamatergic synapse	9.8	<2.0	<2.0	<2.0	2.8	2.9	4.5	<2.0	<2.0
Dopaminergic synapse	9.7	<2.0	2.1	<2.0	<2.0	<2.0	<2.0	<2.0	<2.0
GABAergic synapse	8.0	4.5	<2.0	3.6	<2.0	2.2	<2.0	<2.0	<2.0
Long-term depression	7.5	<2.0	<2.0	<2.0	<2.0	<2.0	<2.0	<2.0	<2.0
Neurotrophin signaling pathway	4.9	<2.0	2.2	<2.0	<2.0	<2.0	<2.0	<2.0	<2.0
Serotonergic synapse	3.0	<2.0	<2.0	<2.0	<2.0	<2.0	<2.0	<2.0	<2.0

Figure S3. KEGG enrichment of DE mRNA for Neuronal System items in 8 types of brain cells.

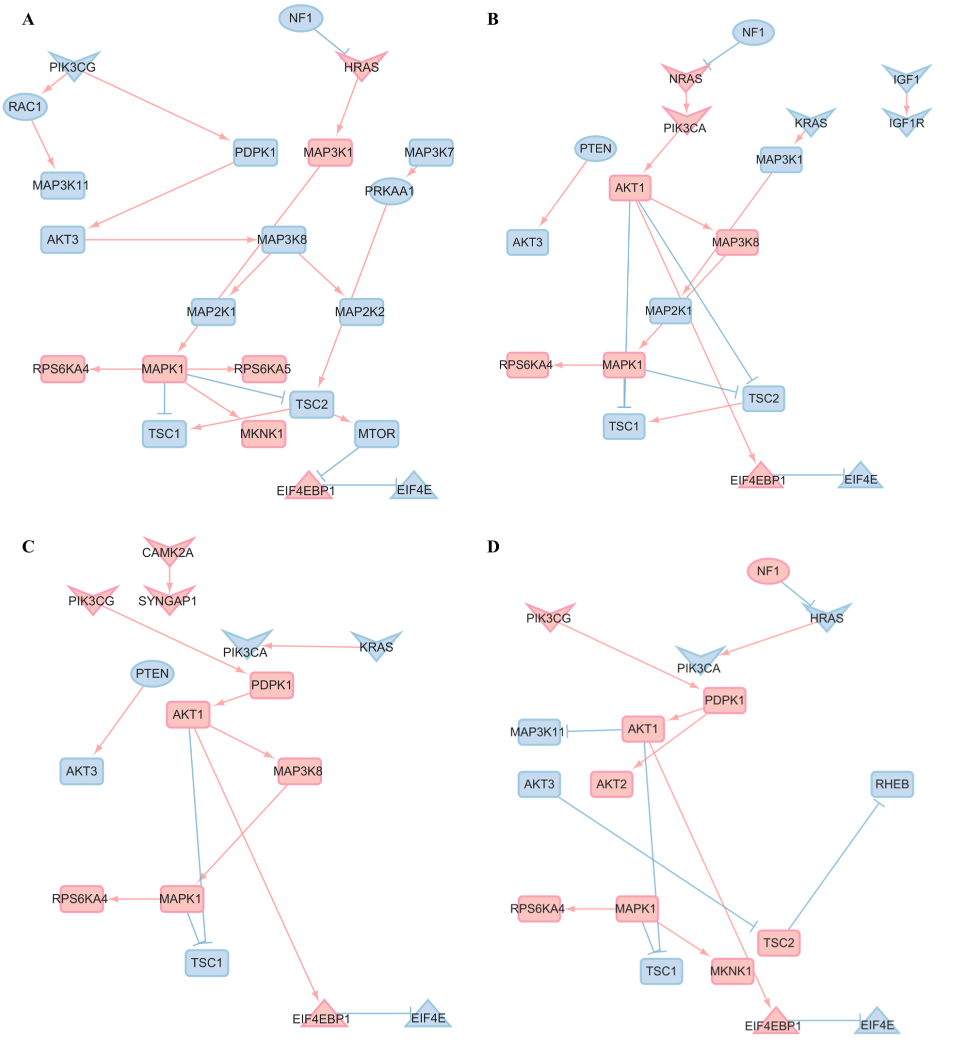

Figure S4. Employing NIVaCaR to identify a set of activated edges and nodes for L neurons within the mSiReN.

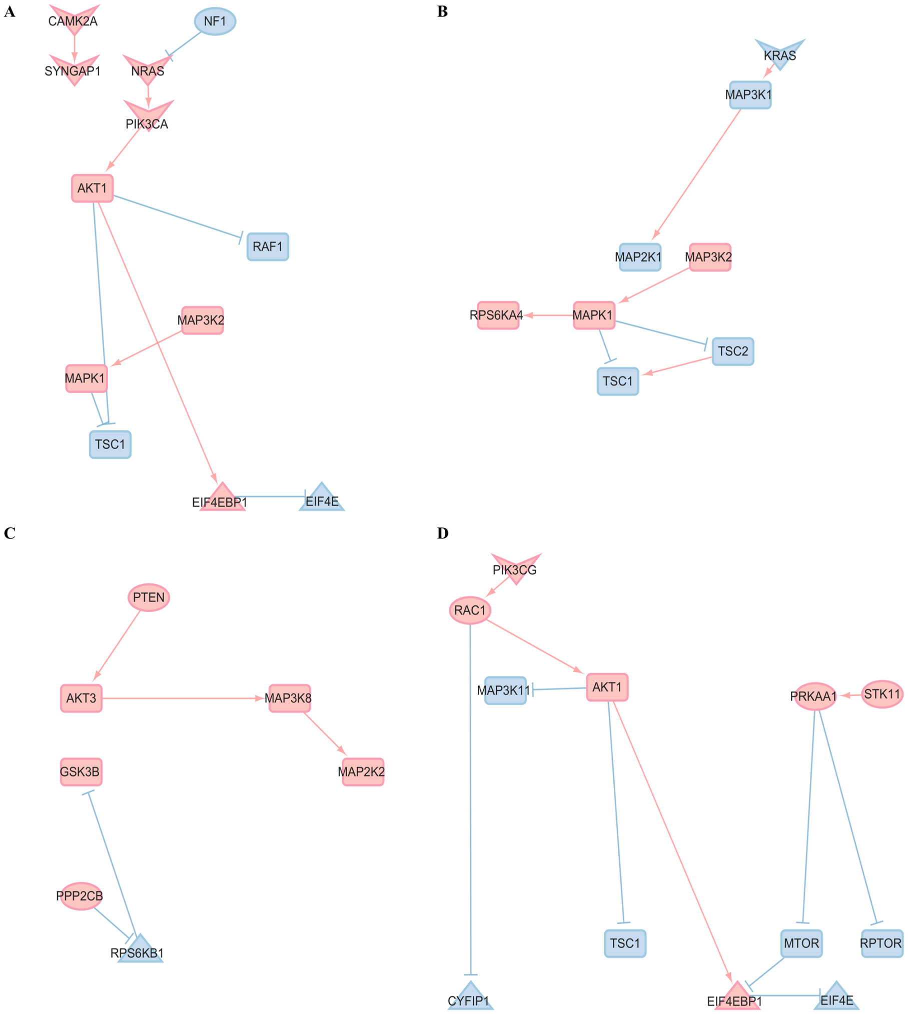

Figure S5. Employing NIVaCaR to identify a set of activated edges and nodes for IN neurons within the mSiReN.

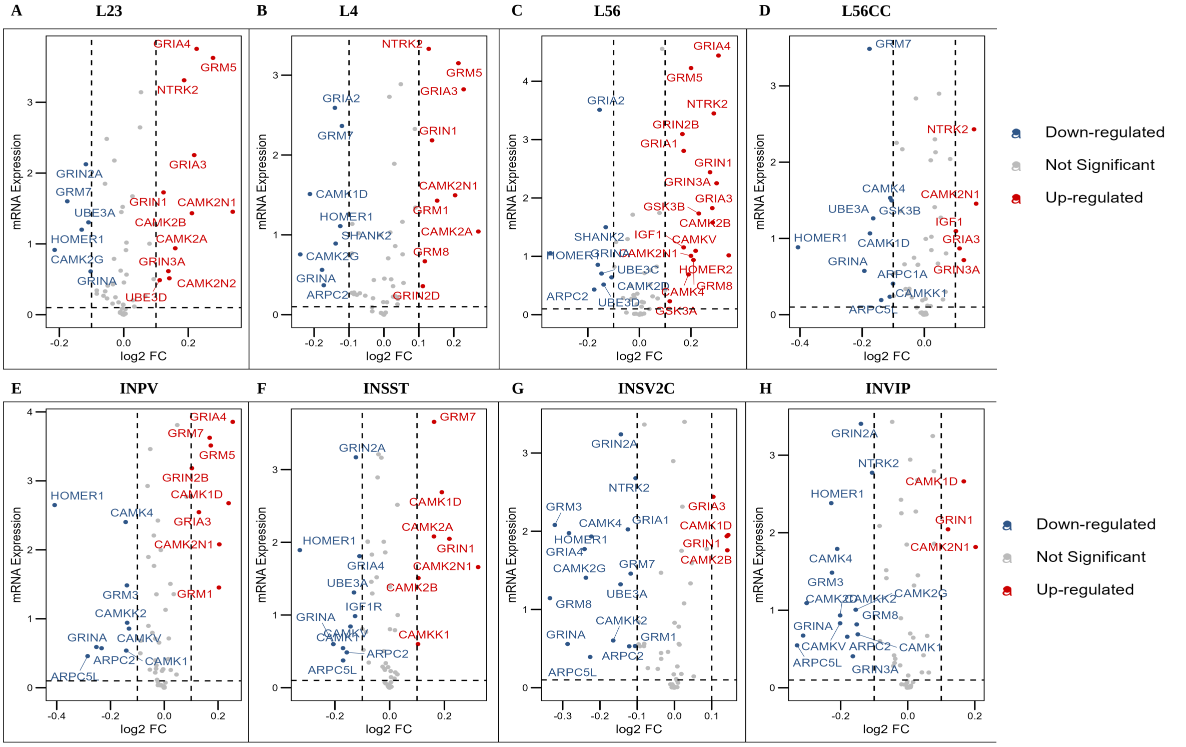

Figure S6. Volcanic map of expression and FC value for translation control related RNA in 8 cell types.

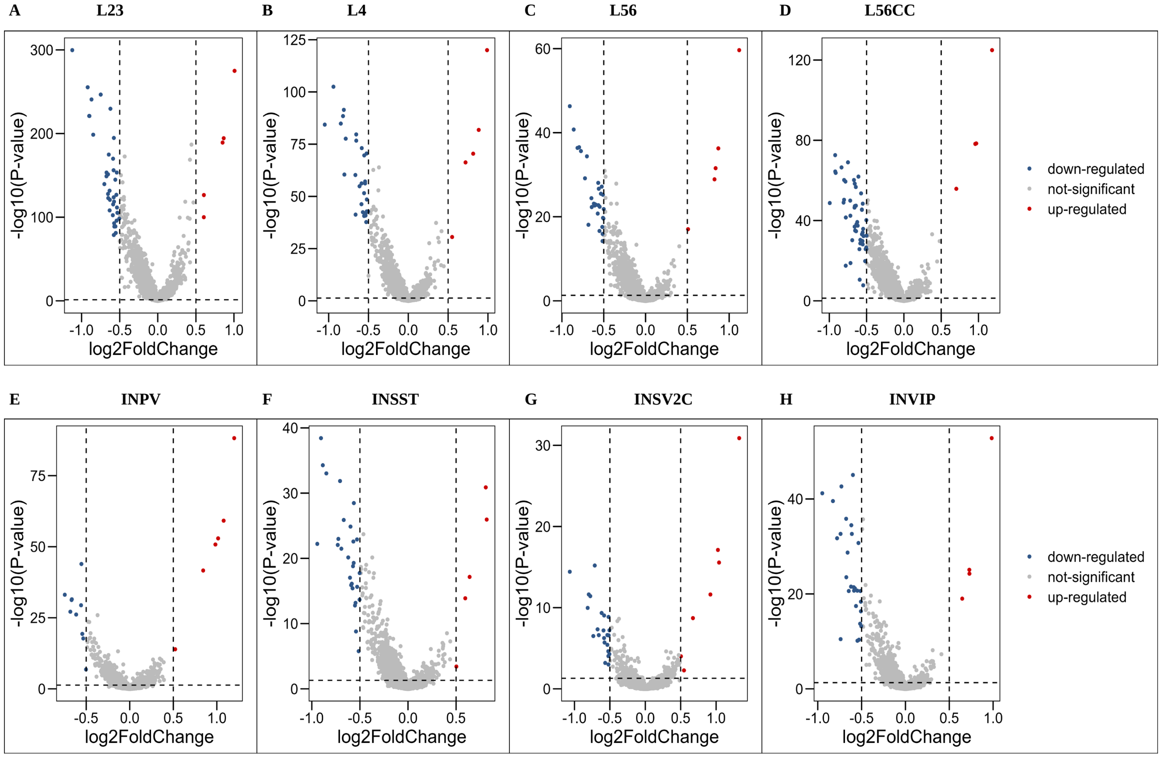

Figure S7. Volcanic map of DE P-Value and FC value for all DE RNAs in 8 cell types.

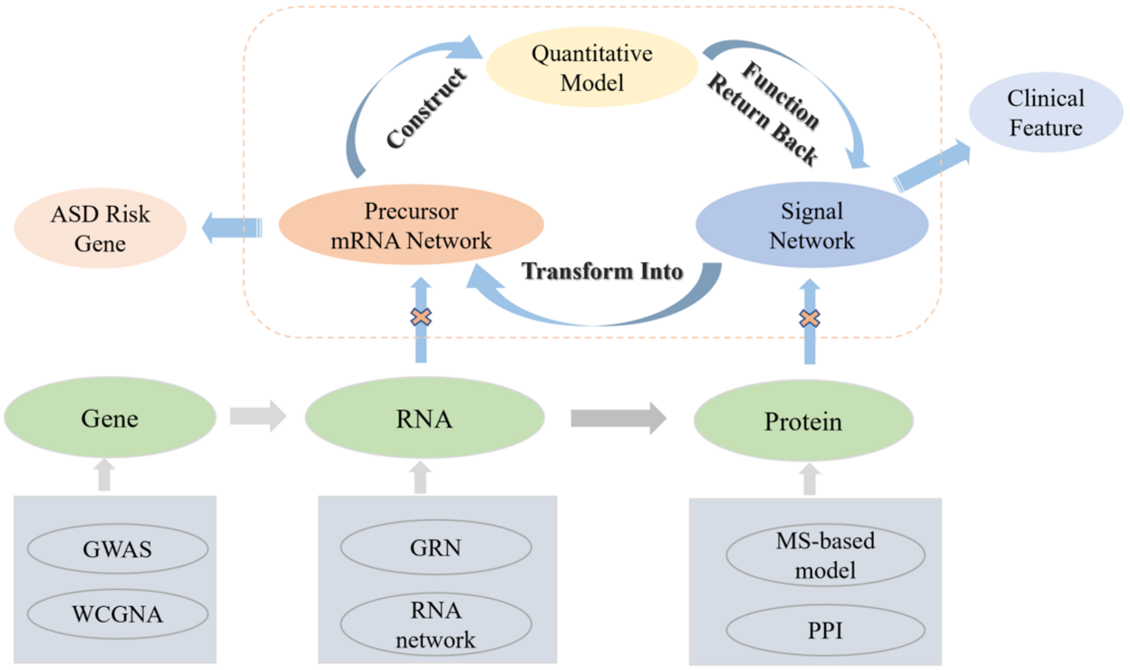

Figure S8. Methodology for Investigating Convergence in ASD Molecular Dysfunction. The molecular convergence mechanism aims to integrate studies at the individual molecular level. Our research combines the signaling network of protein regulation with transcriptional-level regulatory relationships in the context of the disease.

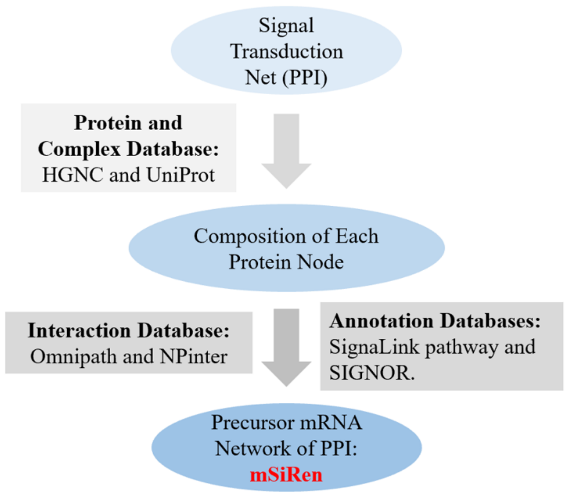

Figure S9. The construction process of mSiReN using databases. We transform the proteins in the signal network into their corresponding precursor mRNAs based on databases HGNC and UniProt. We find the relationship of regulation by interaction databases Omnipath and NPinter and annotation databases SignaLink pathway and SIGNOR.

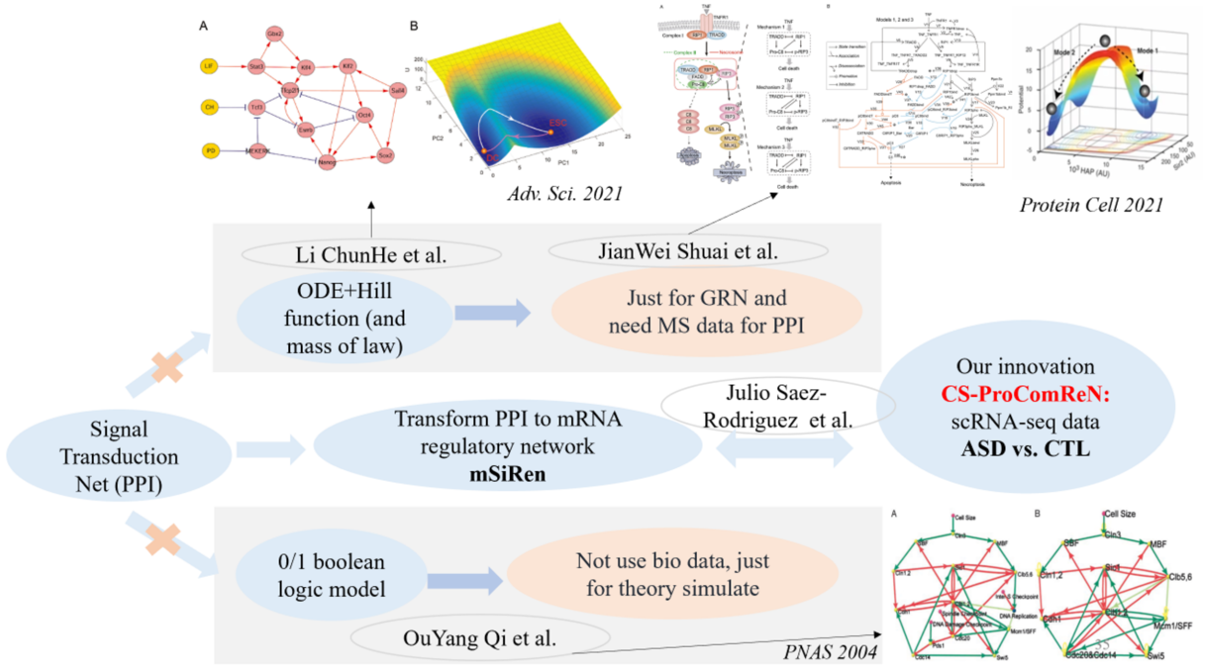

Figure S10. A comparison of the ProComReN method in computational biology. Instead of directly using ODE equations and pure mathematical modeling with a huge number of parameters, our ProComReN model integrates single-cell omics data and prior knowledge networks to investigate the molecular mechanisms of diseases.

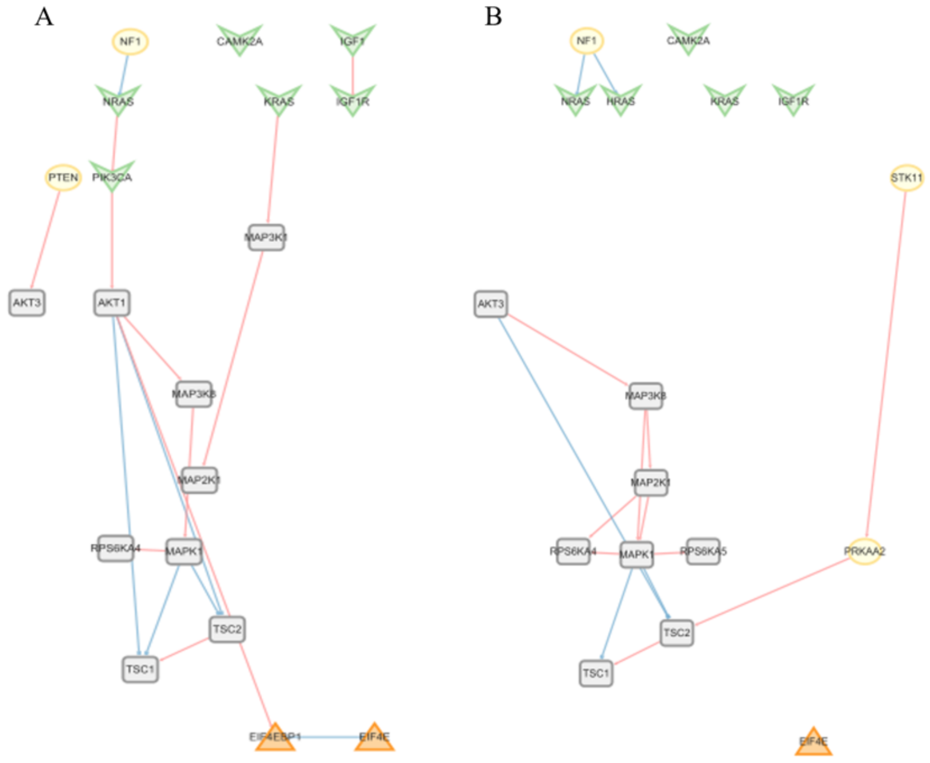

Figure S11. The results of NIVaCaR in L4 and the network of L4 DEGs. A. The signal network is activated by the L4 cell type. B. The signal network of L4 differentiated genes. The fold change of DEGs is larger than 0.01, and the p-value is less than 0.05.

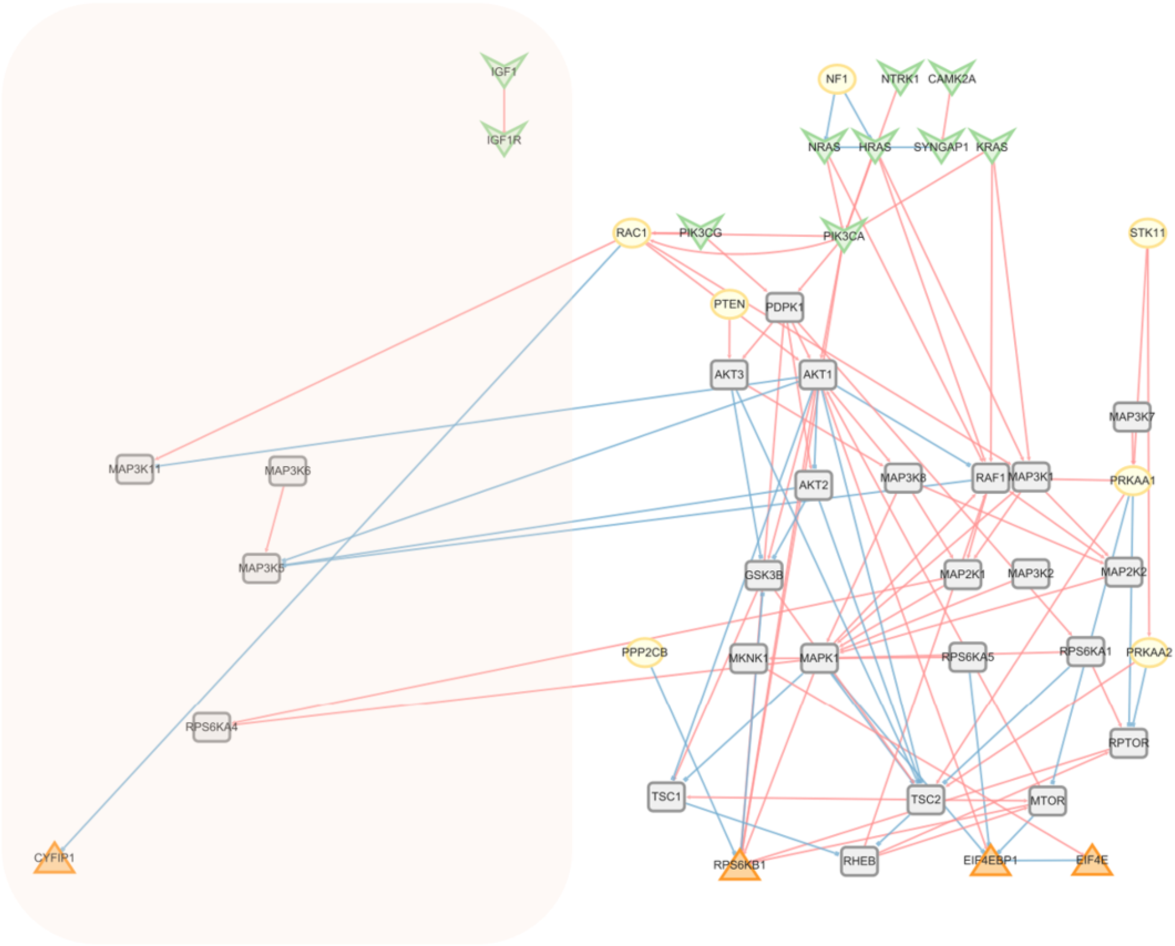

Figure S12. The mSiReN with core module-based inverse trace. Delete some nodes in the pink area that are excluded from the inverse trace. It contains “IGF1”, “IGF1R”, “MAP3K11”, “MAP3K6”, “MAP3K5”, “RPS6KA4”, and “CYFIP1”.

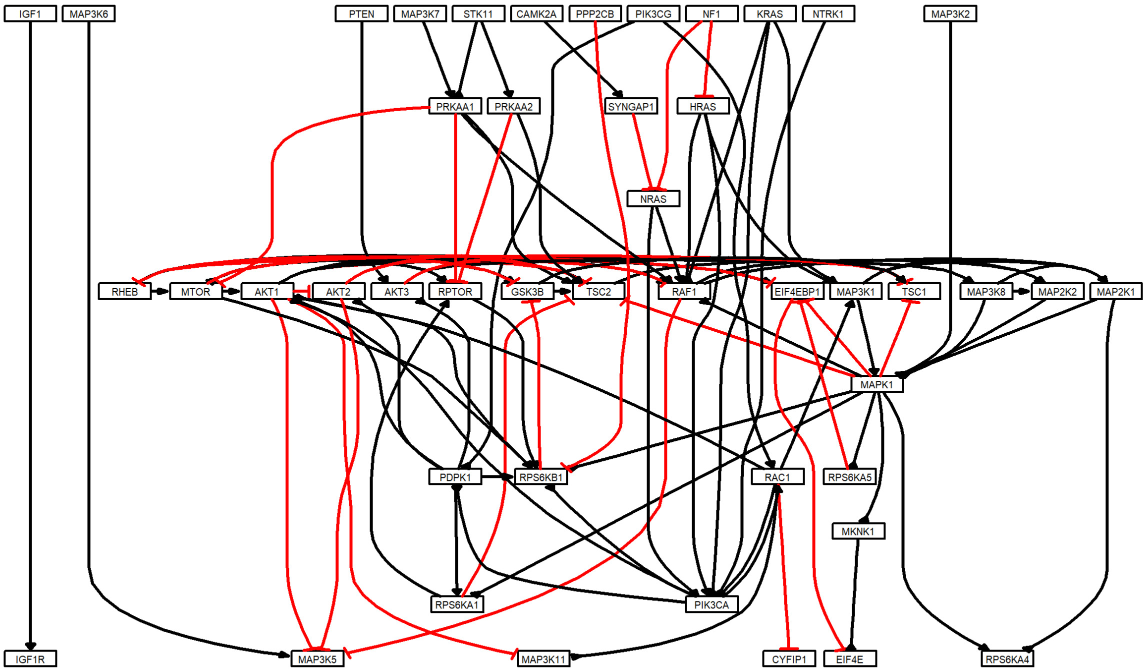

Figure S13. Based on the core module of 4EBP and EIF4E, we filter some nodes from the original network which are excluded from the inverse trace. Account: 7 nodes, ten edges

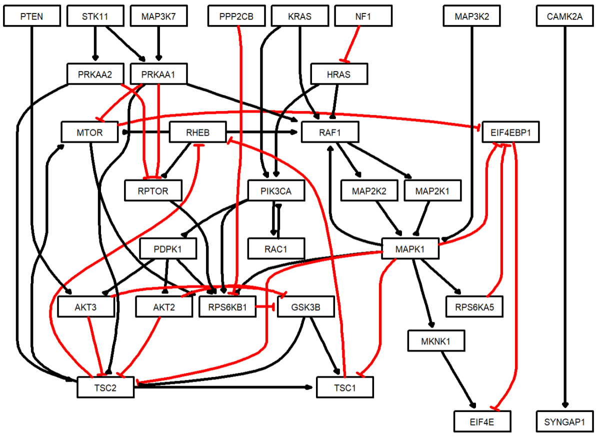

Figure S14. Delete some nodes lower than 20% expression in the cell type.

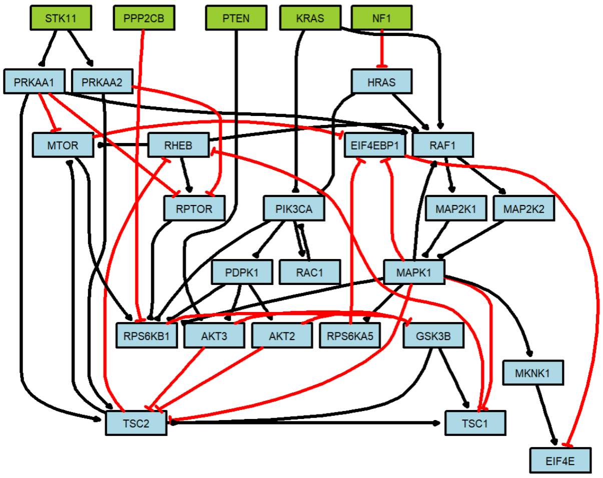

Figure S15. Delete upstream nodes based on topology. Nodes: 28, edges: 53. Input nodes: KRAS, NF1, PPP2CB, PTEN, and STK11.

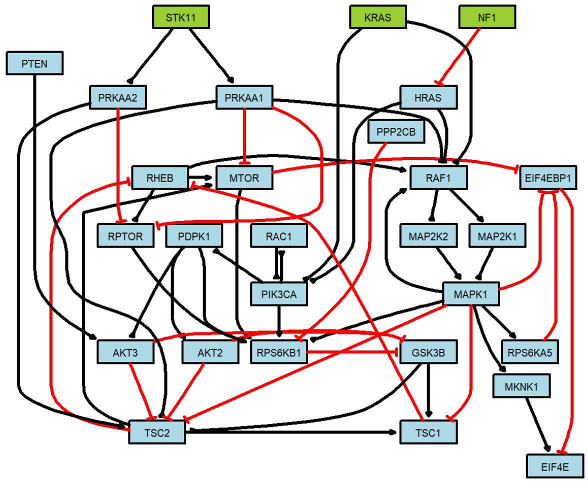

Figure S16. Narrow the network nodes from filtering homolog expression.

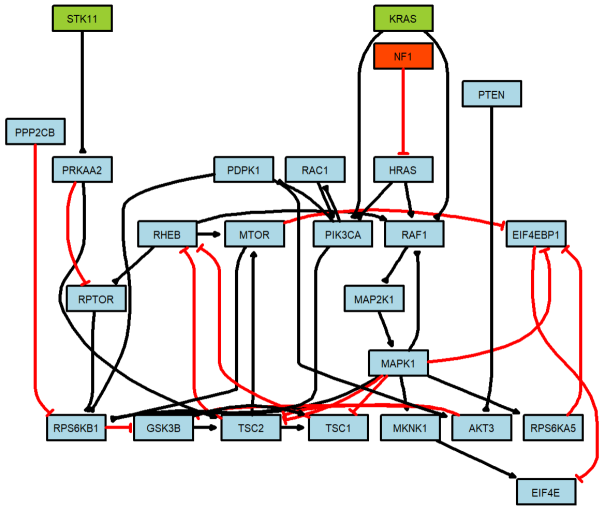

Figure S17. Final network after the reduced preprocess.

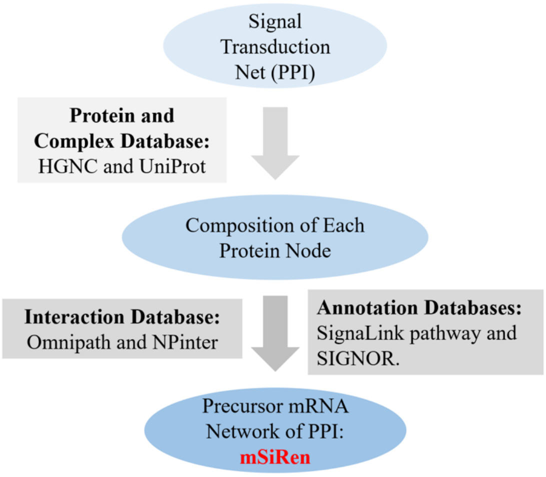

Figure S18. The subnetworks of L4 constructed from NIVaCaR directly.

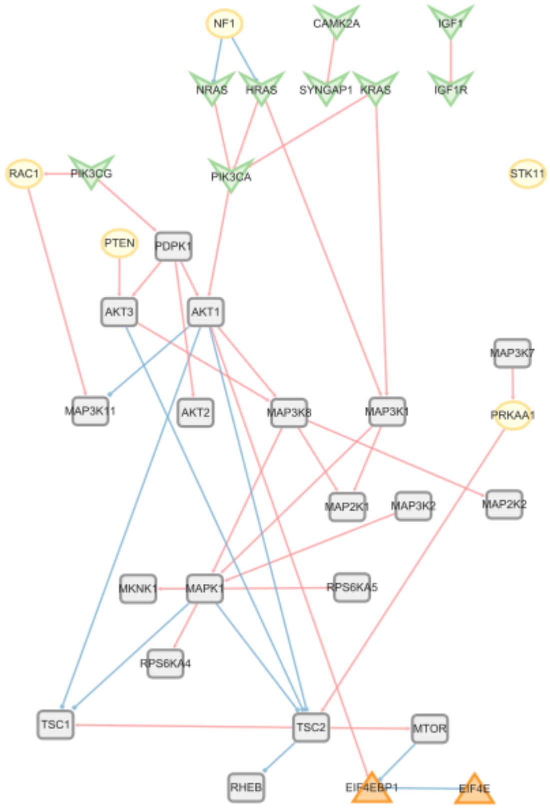

Figure S19. The Union network of L contains 35 nodes and 42 edges.

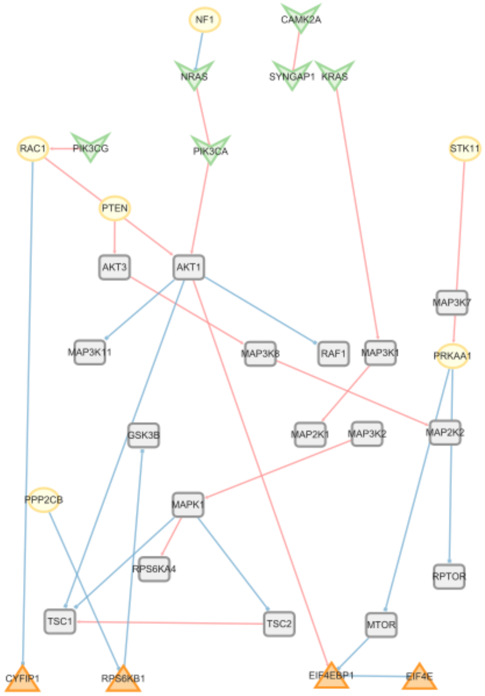

Figure S20. The Union network of IN contains 33 nodes and 28 edges.

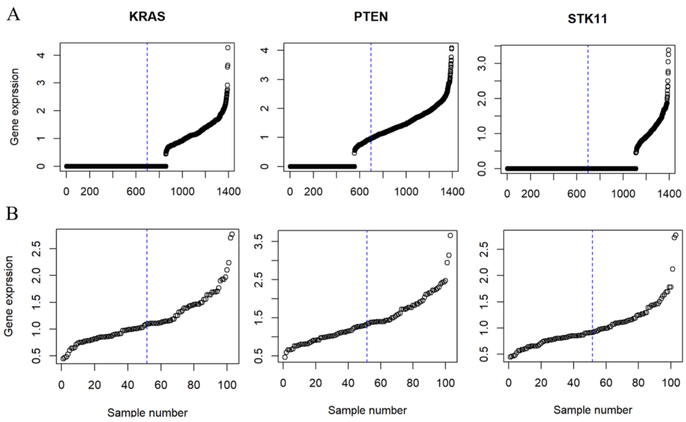

Figure S21. The number of cell in "L" cell types. A. Total "L" types cells sorted according to gene expression. B. All cells with no expression values are discarded from (A).

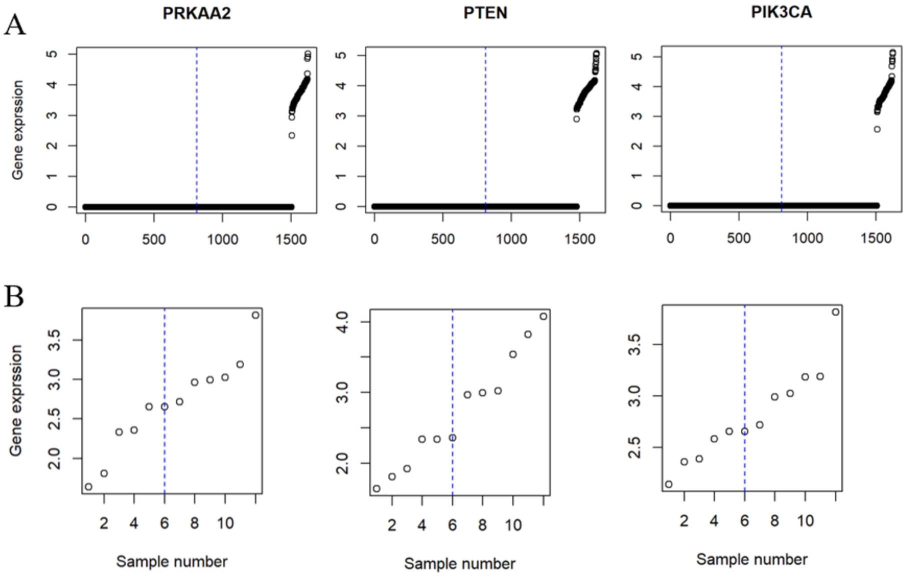

Figure S22. The number of cell in "NEU" cell types. A. Total "NEU" types of cells are sorted according to gene expression. B. All cells with no expression values are deleted from (A).

Supplementary Table

Table S1. Cell number for different cell types in ASD and CTL from the high-throughout scRNA-seq data.

Cell Types	Num. total	Num. ASD	Num. CTL
AST-FB	1571	1001	570
AST-PP	2395	1889	506
Endothelial	991	510	481
IN-PV	1481	829	652
IN-SST	1581	854	727
IN-SV2C	610	417	193
IN-VIP	1908	1289	619
L2/3	5151	3146	2005
L4	3195	2077	1118
L5/6	1281	773	508
L5/6-CC	1562	920	642
Microglia	1027	635	392
Neu-mat	1459	945	514
Neu-NRGN-I	1164	616	548
Neu-NRGN-II	3024	1656	1368
Oligodendrocytes	3749	2055	1694
OPC	3207	1935	1272
Total Num.	35,356	21,547	13,809

Table S2. The numbers of DE RNAs across different cell types from the high-throughout scRNA-seq data in ASD.

Cell Types	Num. DE RNA
AST-FB	145
AST-PP	172
Endothelial	172
IN-PV	56
IN-SST	89
IN-SV2C	96
IN-VIP	53
L2/3	112
L4	86
L5/6	55
L5/6-CC	96
Microglia	167
Neu-mat	64
Neu-NRGN-I	122
Neu-NRGN-II	122
Oligodendrocytes	48
OPC	73

Table S3. Network for source or end nodes in signal transduction network.

	**Label in signal transduction network (PPI and complex)**	**Precursor mRNA (gene)/ regulation**
Source:	"mGlu"	GRM (GRM1, GRM5)
	"iGlu"	GRIA1-4; GRIN1, GRIN2A-D, GRIN3A-B.
	"CAMKII"	CAMK2A
	"BDNF"	NTRK1, NTRK2, NTRK3
	"SYNGAP1"	SYNGAP1
	"IGF1"	IGF1, IGF1R
	"RAS"	HRAS, NRAS, KRAS4A and KRAS4B
	"PI3K"	PIK3CA_PIK3R1, PIK3CA/PIK3CB, PIK3CG
Target	"EIF-4E"	EIF4E
	"4E-BP"	EIF4EBP1, EIF4EBP1
	"S6K1"	RKS6KB1, RKS6KB1, EIF4B/RPS6E

Table S4. Network process 1. Filtering by signal annotation databases.

	**44 Initial nodes**	**Remain nodes**
**Source Nodes** (Receptors and Upstream Signals)	CAMK1, CAMKID, CAMKIG, CAMK2A, CAMK2B, CAMK2D, CAMK2G, CAMK2N1, CAMK2N2, CAMK4, CAMKK1, CAMKK2, GRINIGRM2, GRIN2A, GRIN2B, GRIN2C, GRIN2D, GRIN3A, GRIN3B, GRM1, GRM3, GRM4, GRM5, GRM6, HRAS, IGF1, KRAS, GRM7, GRM8, IGFIR, NRAS, NTRKI, NTRK2, NTRK3, PIK3C2A, PIK3C2B, PIK3C2G, PIK3C3, PIK3CA, PIK3CB, PIK3CD, PIK3CG, SYNGAP1	CAMK2A, HRAS, NTRK3, PIK3CA, IGEl, KRAS, IGEIR, SYNGAPI, PIK3CG, NRAS, NTRK1, NTRK2
**Target Nodes** (Translation Control)	CYEIP1, CYFIP2, EIF4E, EIE4E1B, EIF4E2, EIE4E3, EIF4EBP1, EIF4EBP2, EIF4EBP3, RPS6KB1, RPS6KB2, RPS6KC1, RPS6KL1	EIF4E, EIF4EBP1, RPS6KB1

Table S5. Network process 2. Matching interaction database.

**Nodes remain**	**Nodes discard**
AKTI, AKT2, AKT3, CAMK2A, EIF4E, EIF4EBP1, GSK3B, HRAS, IGF1, IGFIR, KRAS, MAP2K1, MAP2K2, MAP3K1, MAP3K11, MAP3K2, MAP3K5, MAP3K6, MAP3K8, MAPK1, MKNKI, MTOR, NRAS, NTRKI, PDPKI, PIK3CA, PIK3CG, PTEN, RAFI, RHEB, RPS6KA1, RPS6KA4, RPS6KA5, RPS6KB1, RACI, RPTOR, SYNGAP1, TSCl, TSC2	MAP3K10, NTRK2, MAP3K12, MAP3K13, NTRK3, RAC2, MAP3K20, MAP3K3, STK11, MAP3K4, MAP3K7, MAP3K9

Table S6. Network process 3. Adding some super nodes about synapse plasticity.

**Super nodes supplement**	**Final discard super nodes**
ADNP, PP2A, CYFIP1, AMPK, EN2, LKB1 (STK11), FMRP, NE1	ADNP, NMDAR(GRIN1), EN2, NMDAR,(GRIN2A-D), FMRP, mGluR1/5 (GRM1,GRM5)

Table S7. The NIVaCaR variables and their descriptions.

**Variable**	**Description**
$c_{j,k} \in \{-1, 0, 1\}$	Activation/inhibition state of measured species j for cell type k
$x_{j,k} \in \{-1, 0, 1\}$	Predicted activation/inhibition state of species j for cell type k
$x^{+}_{j,k} \in \{0, 1\}$	Potential of node j to be activated for cell type k
$x^{-}_{j,k} \in \{0, 1\}$	Potential of node j to be inhibited for cell type k
$u^{+}_{i,k} \in \{0, 1\}$	Potential of interaction i to activate its target node for cell type k
$u^{-}_{i,k} \in \{0, 1\}$	Potential of interaction i to inhibit its target node for cell type k
$\sigma_{i,k} \in \{-1, 1\}$	Sign of interaction i for cell type k
$d_{j,k} \in \{0, M\}$	Auxiliary distance variables assigned to each node j where M is a sufficiently large number (default: $M = 100$) for cell type k
$A_{j,k} \in \{0, 2\}$	Auxiliary variable representing the absolute difference between the inferred and measured species j for cell type k

Table S8. Nodes filtering from homolog.

Ortholog Symbol HGNC/ Subunit	Node Label in Network	AveExpr
AKT2	AKT (PKB)	0.3607864
AKT3	AKT (PKB)	2.90043385
MAP2K1	MEK1/2(MAP2K)	1.00998264
MAP2K2	MEK1/2(MAP2K)	0.29219968
PRKAA1	AMPK	0.26095921
PRKAA2	AMPK	1.11882331

Table S9. Different types of biological interactions modeled by different Boolean functions and their algebraic representations.

Biological equivalent	Graphical form	Algebraic computation
Activation	$A \rightarrow Z(k)$	$Z_{t+1} = A_t * k$
Inhibition	$A \dashv Z(k)$	$Z_{t+1} = 1 - A_t * k$
Complex formation	$A\ and\ B \rightarrow Z(k)$	$Z_{t+1} = A_t * B_t * k$
Competitive interaction	$A\ or\ B \rightarrow Z(k)$	$Z_{t+1} = 1 - [(1 - A_t) * (1 - B_t) * k]$
Non-competitive interaction	$A \rightarrow Z(k_1)B \rightarrow Z(k_2)$	$Z_{t+1} = A_t * k_1 + B_t * k_2(k_1 + k_2 = 1)$
